# A Prussian Blue Nanozyme‐Adjuvanted Vaccine Presenting Phosphocholine Antigens for Induction of Immunotolerance in Inflammatory Bowel Disease

**DOI:** 10.1002/advs.202522027

**Published:** 2026-01-22

**Authors:** Jingyi Sheng, Yubo Huang, Xinyue Li, Yin Liu, Yuehuang Wu, Yuxin Zhang, Ning Gu

**Affiliations:** ^1^ Jiangsu Key Laboratory for Biomaterials and Devices, School of Biological Sciences & Medical Engineering Southeast University Nanjing China; ^2^ Key Laboratory for Bio‐Electromagnetic Environment and Advanced Medical Theragnostic School of Biomedical Engineering and Informatics Nanjing Medical University Nanjing China; ^3^ School of Chemistry and Chemical Engineering Southeast University Nanjing Jiangsu China; ^4^ Engineering Medicine Research Group and Nanjing Research Center for Biomedical Electron Microscopy (NRC‐BEM) Medical School Nanjing University Nanjing China

**Keywords:** antibody, inflammatory bowel disease, phosphorylcholine, prussian blue nanozymes, vaccine

## Abstract

Inflammatory bowel disease (IBD) is a complex disorder characterized by chronic intestinal inflammation and impaired barrier function, for which effective long‐term therapies remain elusive. To address this challenge, we developed a novel nano‐vaccine that combines Prussian blue nanozymes (PBNZs) adjuvants with a ferritin carrier loaded with phosphorylcholine (PC), an autoimmune‐related antigen (Ferritin‐PC@PBNZs). This vaccine aims to provide sustained protection and therapeutic benefits for IBD through an active immunization strategy. This vaccine leverages the ferritin carrier to effectively present PC antigens, while simultaneously enhancing antigen uptake and activating dendritic cells with the PBNZs adjuvant. This dual action induces a durable humoral immunity characterized by a high‐titer, antigen‐specific antibody response with a balanced Th1/Th2 immune profile. In the dextran sulfate sodium (DSS)‐induced acute colitis model, vaccination with the Ferritin‐PC@PBNZs vaccine markedly alleviated disease severity. This protective effect was attributed to the induction of high levels of specific antibody against inflammation‐associated PC antigen and the restoration of intestinal barrier integrity. Overall, this vaccine‐based immunotolerance therapy represents a promising long‐term therapeutic strategy for IBD.

## Graphical abstract

This study developed a novel nano‐vaccine (Ferritin‐PC@PBNZs) comprising a ferritin‐based scaffold for the multivalent display of phosphorylcholine (PC) antigens, integrated with Prussian blue nanozymes (PBNZs) as a potent adjuvant. By leveraging the ferritin carrier for high‐density and ordered antigen display, the nano‐vaccine significantly augments BCR recognition. PBNZs‐induced lysosomal ROS signaling facilitates DCs maturation and antigen presentation to drive Tcell activation. This synergistic process provides critical T‐cell help, orchestrating the differentiation of B cells into plasma cells for the production of high‐affinity, PC‐specific IgG. This active immunization strategy represents a promising paradigm for the long‐term management of IBD.

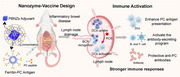

## 1 Introduction

Inflammatory bowel disease (IBD) is a multifactorial, chronic, and non‐specific intestinal inflammatory disorder, with Crohn's disease and ulcerative colitis being the two major clinical subtypes [1, 2]. Although the pathogenesis of IBD remains incompletely understood, it is widely recognized that the disease results from a disruption of intestinal mucosal immune homeostasis in genetically susceptible individuals under the influence of various environmental factors [3, 4, 5]. The central mechanism involves the interaction between impaired intestinal epithelial barrier function and defects in the mucosal immune system [6]. The release of proinflammatory mediators further damages epithelial cells and increases antigen exposure, creating a positive feedback loop that perpetuates chronic, immune‐mediated inflammation [7]. Clinically, symptoms such as fever, persistent diarrhea, abdominal pain, and hematochezia reflect the underlying defects in the intestinal epithelial and mucosal immune systems [8, 9, 10, 11, 12].

Currently, clinical management of IBD primarily relies on long‐term pharmacological interventions aimed at inducing and maintaining clinical remission [13, 14, 15]. These treatments include traditional anti‐inflammatory agents [16, 17, 18], broad‐spectrum immunosuppressants (corticosteroids and azathioprine) [19, 20] as well as more targeted biologics (anti‐TNF‐α monoclonal antibodies and anti‐integrin antibodies) [21, 22, 23] and small molecule drugs such as JAK inhibitors [24, 25, 26]. While these therapies have improved patient outcomes to some extent, they are fundamentally limited by their reliance on long‐term administration [27, 28]. Such a therapeutic approach is heavily dependent on patient adherence and carries significant risks, including an increased susceptibility to opportunistic infections and malignancies due to prolonged systemic immunosuppression [29, 30]. Beyond conventional anti‐inflammatory and immunosuppressive regimens, pathogenic‐factor scavenging has emerged as a biomaterials‐based strategy for IBD intervention. In particular, cell‐free DNA (cfDNA) can act as an inflammatory stimulus, motivating the development of cationic and catalytic “nanoscavengers” that capture and degrade cfDNA to attenuate nucleic‐acid sensor driven inflammation [31]. In contrast, active immunapies designed to reshape immune balance by inducing antigen‐specific immune tolerance offer a promising alternative [32, 33]. These strategies aim to stimulate the body to generate long‐lasting, self‐regulating antibodies, potentially enabling a single intervention to achieve prolonged clinical remission. Therefore, the development of active immunapies that harness the patient's own immune system to produce therapeutic antibodies or cellular responses holds considerable potential for advancing IBD treatment.

Phosphocholine (PC) is a widely recognized antigenic epitope commonly found on apoptotic cells, damaged cell membranes, oxidized lipids, and various pathogens [34, 35]. Notably, clinical observations have revealed a significant positive correlation between elevated levels of natural anti‐PC antibodies and improved disease prognosis in autoimmune and inflammatory disorders, such as systemic lupus erythematosus and atherosclerosis [36, 37, 38]. This suggests that endogenous anti‐PC immune responses play a pivotal role in regulating inflammation and promoting tissue repair. Preclinical studies provide direct evidence supporting this hypothesis, demonstrating that treatment with PC‐conjugated nanofiber peptides effectively alleviates disease severity in mouse models of experimental colitis [39].In DSS colitis, epithelial injury and oxidative stress expose PC‐containing structures on apoptotic or necrotic debris and generate PC‐bearing oxidized phospholipids, which can perpetuate innate immune activation and cytokine amplification. By binding these PC‐positive ligands, anti‐PC antibodies may sequester damage‐associated cues and facilitate their opsonization‐dependent clearance by phagocytes, thereby promoting a pro‐resolving efferocytic programme and dampening inflammatory signalling [39, 40]. By lowering inflammatory burden and accelerating removal of damage‐associated ligands, anti‐PC antibodies are expected to reduce mucosal immune activation, creating conditions that support epithelial restitution and the recovery of tight‐junction architecture. Although the translation of PC‐based therapies into clinical practice faces numerous challenges, current evidence suggests that enhancing the levels of specific anti‐PC antibodies through active immunization strategies may bolster the body's protective immune mechanisms. This approach holds significant potential for the development of innovative therapies for inflammatory bowel disease.

Vaccine formulations are designed to activate the host's adaptive immune response, with adjuvants playing a crucial role in enhancing their efficacy [41, 42]. An effective adjuvant not only facilitates the efficient uptake of antigens but also promotes the maturation and antigen presentation of antigen‐presenting cells (APCs), while modulating the local microenvironment to direct specific immune responses. Alum reliably enhances antibody responses through antigen retention and slow release, yet it often provides limited support for cross‐presentation and cellular immunity and tends to favor Th2‐biased outcomes [43]. Oil‐in‐water emulsions such as MF59 or AS03 rapidly amplify immunity by recruiting innate cells and engaging local inflammatory networks, but their benefit is closely tied to non‐specific inflammatory amplification and increased local reactogenicity [44, 45]. TLR agonists such as CpG or MPLA provide defined pattern recognition receptors (PRR) that potently drive dendritic cell maturation and preferentially promote Th1 and cellular immunity, although they typically require tighter control of dose windows and careful management of systemic inflammatory spillover [46, 47, 48]. In contrast, Prussian blue nanozymes (PBNZs) represent an alternative adjuvant paradigm [49, 50]. PBNZs as functional nanomaterials with multiple enzyme‐like catalytic activities, mimic the functions of peroxidase (POD), superoxide dismutase (SOD), and catalase (CAT), allowing them to effectively modulate different types of reactive oxygen species (ROS) under various microenvironmental conditions [51]. Over the past decades, these catalytic properties have been extensively exploited in the development of immunodiagnostic assays, biosensors, and anticancer therapeutics [52]. Notably, PBNZs exhibit pronounced POD‐like catalytic activity under acidic conditions, enabling localized modulation of H_2_O_2_ and ROS‐enriched inflammatory cues, remodeling the antigen‐processing microenvironment, and providing endogenous pro‐priming signals. By coupling nanoscale delivery with catalytic signaling, PBNZs can enhance dendritic cells (DCs) uptake, maturation, and migration, improve antigen processing and presentation, promote cross‐presentation and robust cellular immunity, and support a Th1 and Th2 compatible mixed trajectory [53, 54, 55]. In addition, a potential advantage of PBNZs is that their immunostimulatory effects are more likely to be localized and context dependent, enabling immune potentiation without relying on high‐intensity PRR agonism and thereby mechanistically reducing the risk of systemic cytokine surges and associated systemic adverse responses. Overall, PBNZs as an immunological adjuvant can address key limitations of conventional platforms by overcoming constrained antigen‐presentation modes and enabling a more balanced Th1/Th2 immune programming.

Our recent study demonstrated a size‐dependent intracellular enzymatic activity of PBNZs, showing that smaller particles (approximately 3 nm) are more efficient at scavenging ROS upon escaping the lysosomal compartment, while larger particles (60 and 170 nm) retain POD‐like activity within lysosomes, thereby generating higher oxidative stress [56]. During intraperitoneal immunization, internalized antigens are primarily trafficked to the acidic lysosomal compartments of DCs for processing and presentation. In this process, the efficiency of antigen uptake, lysosomal trafficking, and DCs maturation are critical factors determining the magnitude of downstream immune responses. Additionally, intracellular oxidative stress has been identified as key signaling mediator governing DCs maturation [57]. Based on this mechanism, we hypothesize that larger PBNZs can enhance antigen uptake and trafficking to the lysosomes, where their POD‐like activity within the acidic lysosomal environment modulates oxidative stress, thereby promoting DCs maturation and improving their capacity to activate antigen‐specific Th cells.

**SCHEME 1:**
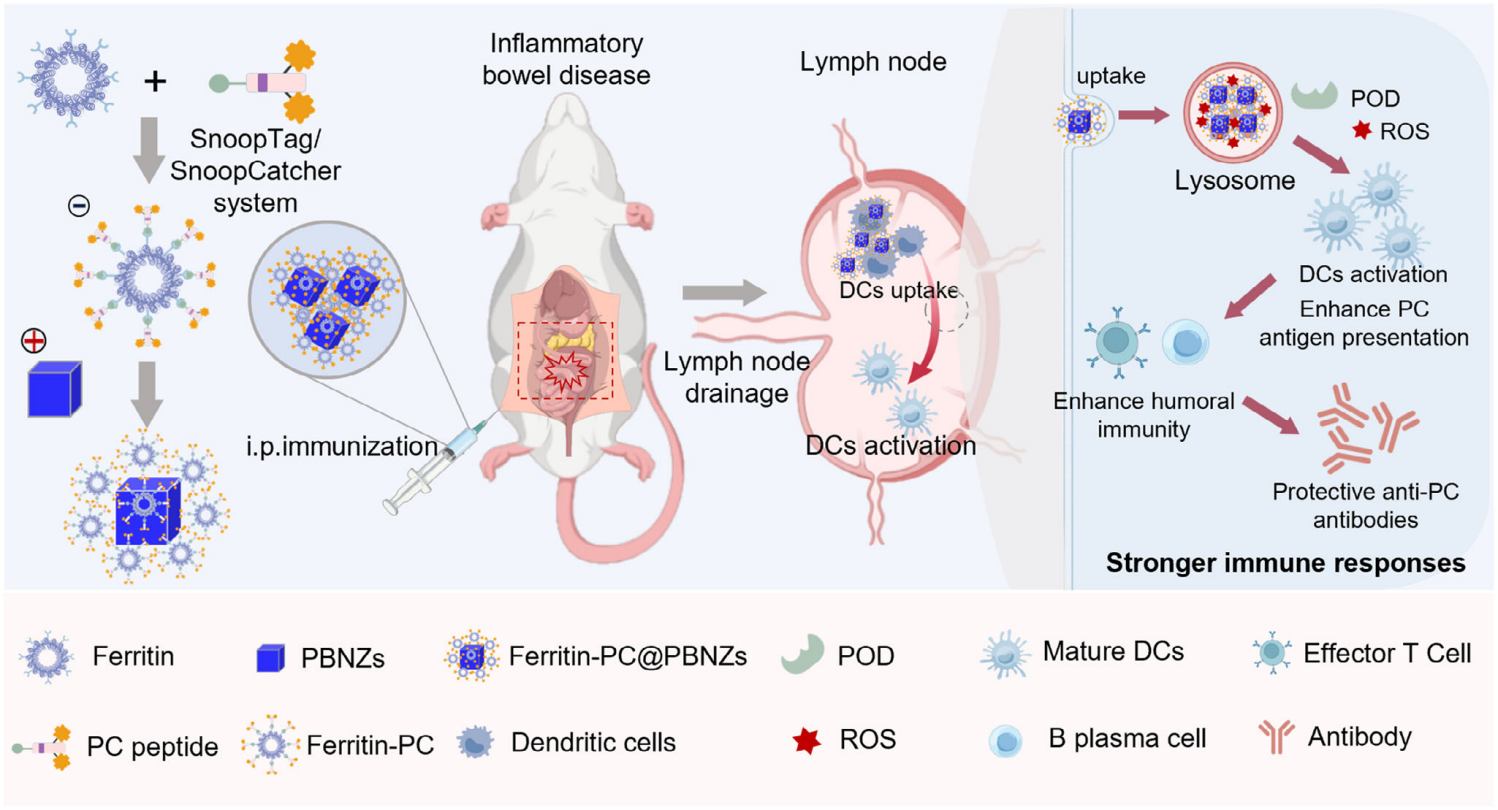
Schematic illustration of the Prussian blue nanozyme (PBNZs) Ferritin‐PC@PBNZs vaccine. Upon internalization by dendritic cells (DCs), the Ferritin‐PC@PBNZs complex localizes to lysosomal compartments where it exhibits peroxidase (POD)‐like activity, generating reactive oxygen species (ROS) that promote DCs maturation. This process enhances the sustained presentation of ferritin‐displayed phosphocholine (PC) antigens, ultimately potentiating robust T‐cell and B‐cell activation for effective immune protection.

Herein, we established a nanozyme‐adjuvanted vaccine platform designed to induce a durable antibody response, therapeutically modulating intestinal inflammation and alleviating IBD (Scheme 1). By harnessing the anti‐inflammatory properties of naturally occurring PC‐specific antibodies, we utilized a ferritin‐based self‐assembled nanocarrier displaying both PC and T helper (Th) epitope to stimulate a lasting PC‐specific IgG antibody response. Moreover, PBNZs adjuvant enhances local antigen density, thereby facilitating more efficient B cell receptor (BCR) recognition and antigen uptake by DCs. Simultaneously, PBNZs promote the activation and functional maturation of DCs, providing essential activation signals that trigger a robust and enduring humoral immune response. The resulting nanozyme vaccine (Ferritin‐PC@PBNZs) markedly enhanced the production of anti‐PC antibodies, including IgM and IgG, effectively preserved intestinal barrier integrity, and exhibited potent therapeutic efficacy in alleviating IBD symptoms. This approach holds great promise as a durable immunotolerance strategy for the treatment of IBD.

## 2 Results and Discussion

### 2.1 Construction of a Ferritin‐Based Vaccine Adjuvanted with PBNZs

**FIGURE 1:**
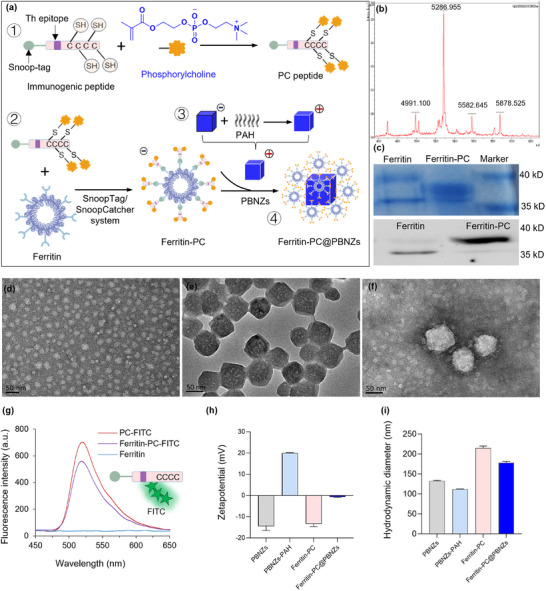
Synthesis and Characterization of Ferritin‐PC@PBNZs. (a) Schematic illustration of the Ferritin‐PC@PBNZs preparation process. (b) MALDI‐TOF mass spectrometry of PC‐peptide conjugates. PC peptide showing the conjugation of 1–4 PC molecules, expected m/z of: PC_1_ = 4991.100, PC_2_ = 5286.955, PC_3_ = 5582.645, and PC_4_ = 5878.525. (c) Coomassie blue staining (top panel) and Western blot analysis (bottom panel) of Ferritin and Ferritin‐PC revealed distinct protein band patterns. (d) Negative‐staining TEM images of Ferritin‐PC. Scale bar: 50 nm. (e) TEM images of PBNZs. (f) Negative‐staining TEM images of Ferritin‐PC@PBNZs. (g) Fluorescence emission spectrum of Ferritin‐PC with FITC‐labeled. *λ*ex = 430 nm, fluorescence collected at 450–650 nm. (h) Zeta potential of PBNZs, PBNZs‐PAH, Ferritin‐PC and Ferritin‐PC@PBNZs nanoparticles. (i) Hydrodynamic size distribution of PBNZs, PBNZs‐PAH, Ferritin‐PC, Ferritin‐PC@PBNZs nanoparticles.

We developed a ferritin‐based nanovaccine in which PC antigen fragments and Th epitope are displayed on the ferritin surface, followed by electrostatic assembly with PBNZs adjuvants. This hierarchical design enables precise antigen presentation and catalytic immune modulation, offering a potent therapeutic strategy against inflammatory bowel disease (Figure 1a). For the antigenic component, the chemically synthesized small‐molecule phosphocholine was covalently conjugated to a custom‐designed short peptide (Synthesis S1). The peptide sequence contained a tetanus toxin–derived Th epitope, multiple cysteine residues with exposed thiol groups serving as active sites for PC modification, and a SnoopTag fragment for subsequent covalent attachment (Figure 1a, ①). Matrix‐assisted laser desorption/ionization time‐of‐flight (MALDI‐TOF) mass spectrometry confirmed the successful conjugation of PC molecules to the peptide, with each peptide chain binding one to four PC molecules, most commonly two (Figure 1b). We ensured that the number of PC moieties per peptide remained as uniform as possible to achieve consistent antigen presentation.

**FIGURE 2:**
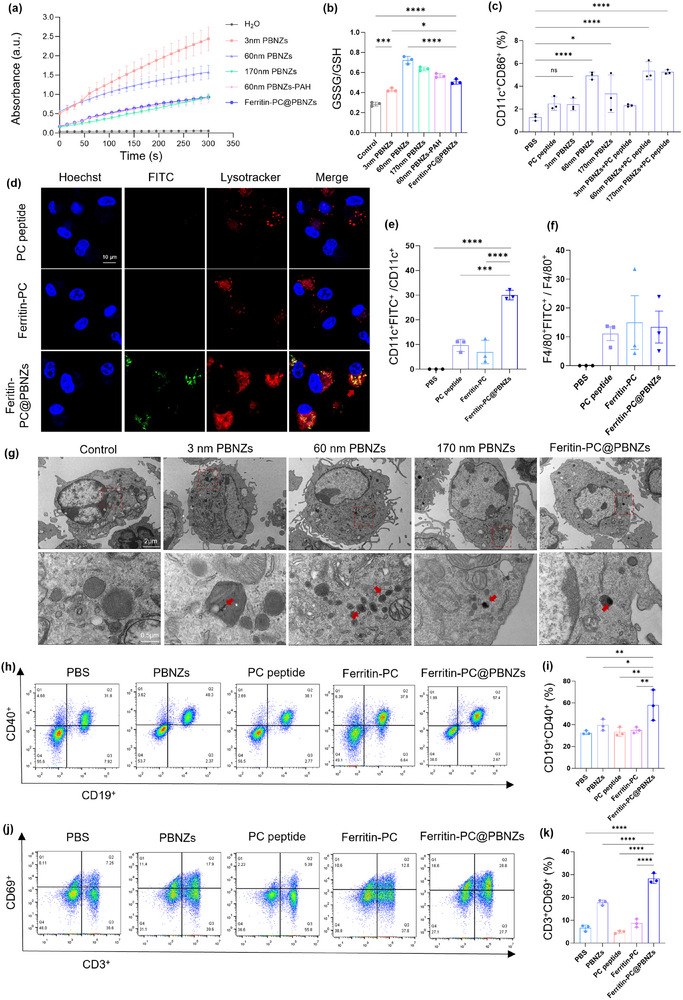
Ferritin‐PC@PBNZs promote DCs maturation and activate T and B cell immunity. (a) POD‐like activity of PBNZs with different sizes and functional modifications at pH 4.6. (b) Intracellular GSSG/GSH ratio in BMDCs after 24 h incubation with the indicated formulations (*n* = 3). (c) Flow cytometric (FCM) quantification of the percentage of CD11c^+^CD86^+^ within among gated lymphocytes. Mice were intraperitoneally injected with PBNZs alone or a physical mixture of PBNZs and PC peptide. After 48 h, cells from intestinal lymph nodes were harvested and analyzed by flow cytometry following staining with anti‐CD11c and anti‐CD86 antibodies. (d) Ferritin‐PC@PBNZs is internalized by DCs and accumulates in lysosomes. Blue: Hoechst 33342. Red: Lysotracker. Green: FITC‐labeled nanomaterials, including PC peptide, Ferritin‐PC, and Ferritin‐PC@PBNZs. Primary DCs were isolated from mouse bone marrow and cultured. Scale bar: 10 µm. (e) FCM quantification of the percentage of CD11c^+^FITC^+^. (f) FCM quantification of the percentage of F4/80^+^FITC^+^. (g) TEM images of BMDCs phagocytosing PBNZs with different sizes and functional modifications. Scale bar: 2 µm (top panel) and 0.5 µm (bottom panel). (h,i) FCM analysis of B cells (h) and quantification of the percentage of CD19^+^CD40^+^ (i) within among gated lymphocytes. (j,k) FCM analysis of T cell (j) and quantification of the percentage of CD3^+^CD69^+^ (k) within among gated lymphocytes. Note: In Figure(h–k), mice were intraperitoneally injected with PC peptide, PBNZs (60 nm size), Ferritin‐PC, or Ferritin‐PC@PBNZs (PBS as control). After 48 h, intestinal lymph node cells were isolated and analyzed by flow cytometry using fluorescently‐conjugated antibodies against anti‐CD11c, anti‐F4/80, anti‐CD19, anti‐CD40, anti‐CD3, and anti‐CD69. *n* = 3 mice. All PC peptides utilized in these analyses were FITC‐conjugated. Differences were considered statistically significant when *p* < 0.05 (^*^), *p* < 0.01 (^**^), *p* < 0.001 (^***^), *p*<0.0001 (^****^).

**FIGURE 3:**
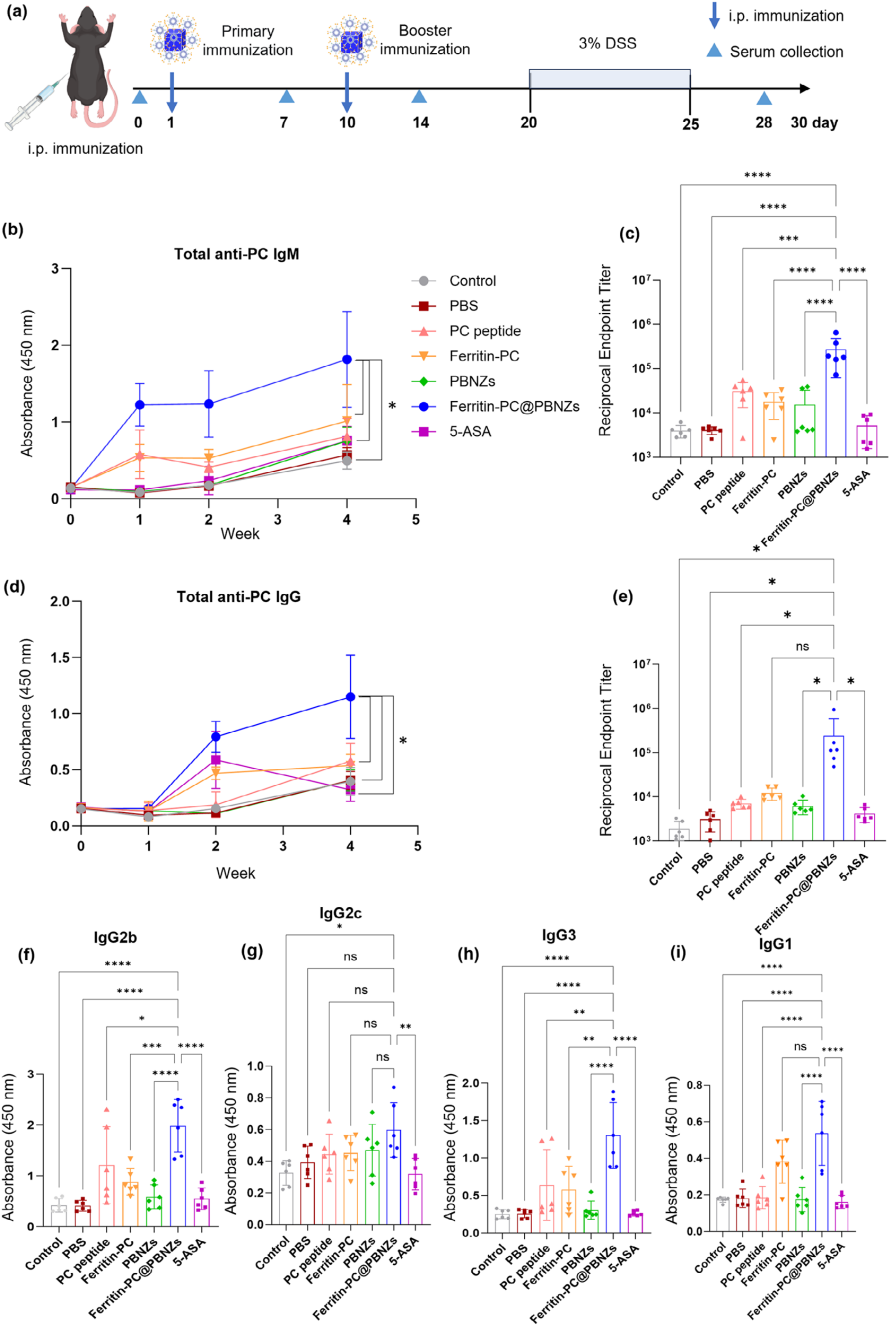
Vaccination with Ferritin‐PC@PBNZs induced a systemic immune response and significantly enhanced antibody levels. (a) Scheme of immunization and sampling. (*n* = 6 per group). (b) Serum total anti‐PC IgM responses. (c) Anti‐PC IgM antibody titers in serum at week 4. (d) Serum total anti‐PC IgG responses. (e) Anti‐PC IgG antibody titers in serum at week 4. (f–i) Serum anti‐PC IgG subclass responses at week 4, including IgG2b (f), IgG2c (g), IgG3 (h), and IgG1 (i). Mean +,—or ± s.e.m. are shown. Statistical significance was determined by two‐way RM ANOVA with Tukey's multiple comparison test for (b) and (d); and one‐way ANOVA with Tukey's multiple comparison test for (c) and (e–i). *p* < 0.05 (^*^), *p* < 0.01 (^**^), *p* < 0.001 (^***^), *p*<0.0001 (^****^), ns, not significant.

**FIGURE 4:**
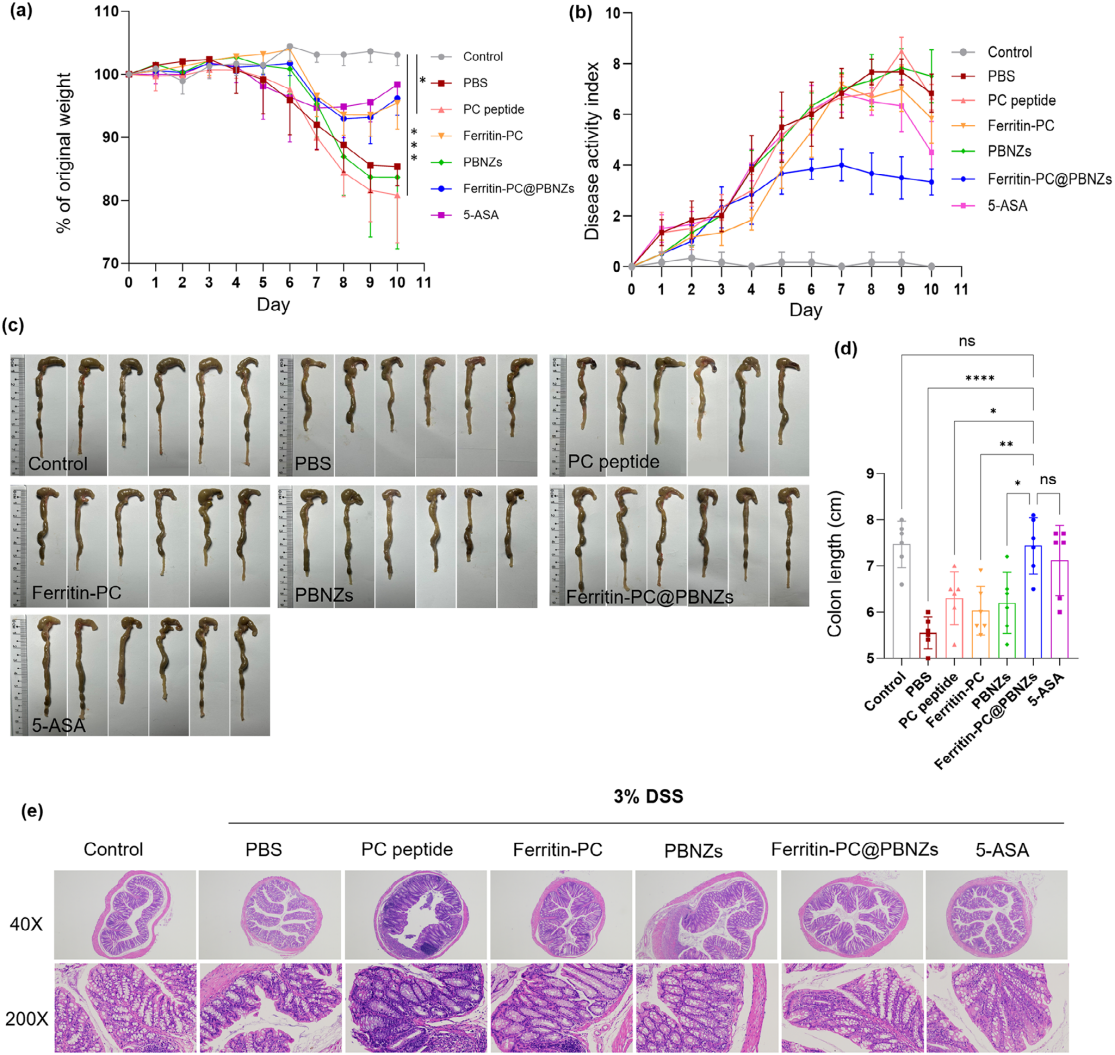
Immunization with Ferritin‐PC@PBNZs is protective in a model of acute colitis. (a) Daily mouse weights as a percentage of their original weights, recorded from one day before DSS treatment to the end of the study. (b) DAI scores as a combination of weight loss, fecal score, and occult blood. (c,d) The picture of colons (c) and the statistical data of colon length (d) of mice in different groups after the mice were sacrificed on day 30. (e) The H&E staining of cross‐sections of the colons. Mixed‐effects analysis with Tukey's multiple comparison test for (a) and (b). Mixed‐effects analysis was used for the longitudinal (repeated‐measures) body weight and DAI data, with treatment and time as fixed effects and individual mice as a random effect; Tukey's multiple‐comparison test was applied for post hoc group comparisons. One‐way ANOVA with Tukey's multiple comparison test for (d). *n* = 6 mice, *p* < 0.05 (^*^), *p* < 0.01 (^**^), *p* < 0.001 (^***^), *p*<0.0001 (^****^), ns, not significant.

Subsequently, the PC‐modified antigenic peptide was covalently linked to the ferritin carrier via the SnoopCatcher/Tag protein conjugation system [58, 59]. This bio‐orthogonal coupling relies on the engineered SnoopTag module on the peptide and the SnoopCatcher domain specifically introduced onto ferritin, enabling rapid and site‐specific assembly of multifunctional nanocarriers (Figure 1a, ②). The sequence information for these constructs is provided in Figure S1. The resulting fusion protein, termed Ferritin‐PC, was purified using immobilized metal affinity chromatography (IMAC), and successful conjugation was confirmed by SDS‐PAGE analysis (Figure 1c). Transmission electron microscopy (TEM) further verified the structural uniformity of Ferritin‐PC (Figure 1d), revealing monodisperse, spherical nanoparticles. Based on our previously study, Prussian blue nanoparticles with an average diameter of approximately 60 nm were selected as the adjuvant due to their prolonged lysosomal residence and a pronounced POD‐like activity as compared with PBNZs of sizes [56]. To ensure a positive surface charge, polyallylamine hydrochloride (PAH) was employed to modify the surface potential of the nanoparticles (Figure 1a, ③). Finally, the negatively charged Ferritin‐PC was electrostatically adsorbed onto positively charged PBNZs to form the complete nanozyme vaccine complex, designated as Ferritin‐PC@PBNZs(Figure 1a, ④). This structure displays a high density of designed antigens on its surface, thereby facilitating more efficient recognition by BCRs and antigen uptake by DCs. TEM revealed that the antigen‐loaded ferritin nanoparticles Ferritin‐PC were stably adsorbed onto the surface of PBNZs without altering their original morphology (Figure 1e,f and Figures S2 and S3). In addition, fluorescein isothiocyanate (FITC) was introduced into the antigenic peptide as a fluorescent tag. Both Ferritin‐PC and Ferritin‐PC@PBNZs exhibited characteristic fluorescence emission peaks at approximately 520 nm, confirming the successful conjugation of Ferritin‐PC@PBNZs (Figure 1g). Zeta potential analysis showed that the polyallylamine hydrochloride modification imparted a positive surface charge to the PBNZs, whereas the assembled Ferritin‐PC@PBNZs complex exhibited a slightly negative surface potential after binding with Ferritin‐PC (Figure 1h). Dynamic light scattering (DLS) measurements further confirmed that all synthesized nanoparticles displayed excellent dispersibility in aqueous solution (Figure 1i). All formulations showed only modest fluctuations in hydrodynamic diameter over 0–7 days at 4°C in PBS containing 1% BSA (pH 7.4), with no apparent aggregation, indicating good colloidal stability under low‐temperature conditions (Figure S4a). By contrast, at 37°C the ferritin‐based systems (Ferritin‐PC and Ferritin‐PC@PBNZs) displayed a marked size increase within 24 h that continued thereafter, consistent with temperature‐driven structural rearrangements in ferritin (Figure S4b). Notably, zeta potentials remained largely unchanged at both temperatures, suggesting that the size evolution did not materially perturb surface charge or interfacial electrostatics (Figure S4c, Figure 4d).

### 2.2 POD‐Like Activity of PBNZs Formulations

To establish a mechanistic rationale for deploying PBNZs as vaccine adjuvants, we focused on their catalytic capacity as an upstream driver of immune programming. POD‐like catalysis can reshape peroxide‐rich inflammatory cues and generate ROS‐based signals, which have been implicated in promoting DCs maturation and enhancing antigen processing and presentation. We therefore quantified POD‐like activity across PBNZ formulations and linked these cell‐free readouts to intracellular redox responses in bone marrow–derived dendritic cells (BMDCs). In cell‐free assays at pH 3.6, PBNZs exhibited strong POD‐like activity that was clearly dependent on both particle size and surface modification (Figure 2a). Relative to 60 nm PBNZs, PAH functionalization reduced the apparent catalytic output, consistent with partial masking of reactive sites and restricted substrate access at the nanozyme interface. Importantly, intrinsic activity measured in solution did not directly translate into intracellular performance. Although 3 nm PBNZs showed the highest POD‐like activity in vitro, they produced only a limited redox shift in BMDCs, as reflected by a modest change in the intracellular GSSG/GSH ratio (Figure 2b). In contrast, larger PBNZs elicited a more pronounced increase in GSSG/GSH, indicating more effective catalytic engagement in the cellular context. This divergence is plausibly explained by size‐dependent differences in phagocytic uptake and intracellular trafficking, which determine nanozyme residence within acidic endo/lysosomal compartments where peroxidase‐like catalysis is favored. Flow cytometry was further used to assess BMDCs activation following exposure to PBNZs of different sizes. Across all formulations, PBNZ treatment increased the frequencies of CD11c^+^CD86^+^ and CD11c^+^MHC II^+^ double‐positive cells, consistent with enhanced DCs maturation (Figure S5). Notably, 3 nm PBNZs elicited a slightly higher activation level than the 60 and 170 nm PBNZs, suggesting that size‐dependent immunostimulation may not be fully explained by POD‐like catalysis alone and could involve additional pathways. Nevertheless, these data collectively support a size‐dependent effect of PBNZs in promoting DCs maturation.

### 2.3 PBNZs Adjuvant Activates Dendritic Cells In Vivo

To explore the potential mechanism of nanozyme adjuvant‐induced immune activation, we examined the activation levels of APCs following in vivo stimulation with various sizes of PBNZs. A mixture of PBNZs and PC peptides was administered intraperitoneally to mice, and after 48 h, intestinal lymph node cells were harvested. Flow cytometry (FCM) was used to assess the activation of DCs (CD11c^+^MHC II^+^/CD86^+^), B cells (CD19^+^CD40^+^), and T cells (CD3^+^CD69^+^). FCM analysis of DCs showed that, compared with the control group, the 60 nm PBNZs‐adjuvanted group exhibited a significantly increased proportion of **CD11c^+^CD86^+^** double‐positive cells, indicating enhanced DCs maturation and antigen‐presenting capacity (Figure 2c; Figure S6). Meanwhile, the proportion of CD11c^+^MHC II^+^ cells exhibited an upward trend, but the overall increase was more modest than that of CD86 (Figure S7a,b). This discrepancy likely reflects asynchronous regulation during in vivo DCs maturation: co‐stimulatory molecules respond rapidly to inflammatory signals, whereas MHC II dependent antigen processing and presentation are limited by antigen load, trafficking kinetics, and local feedback. Meanwhile, FCM analysis of B‐ and T‐cell revealed varying degrees of enhanced activation in the PBNZ‐treated groups, as evidenced by increased frequencies of CD19^+^CD40^+^ B cells (Figure S8a) and CD3^+^CD69^+^ T cells (Figure S8b), indicating that the phenotypic maturation of DCs is functionally transmitted to downstream adaptive immune cells. Collectively, 60 nm PBNZs achieve a more favorable balance between cellular uptake efficiency and lymph‐node delivery, thereby promoting DCs maturation in vivo in a mild yet effective manner and concomitantly enhancing B‐ and T‐cell activation. Therefore, the 60‐nm PBNZs were selected as the baseline material for subsequent experiments.

To verify our hypothesis that internalized PBNZs exert POD‐like activity within lysosomes to modulate oxidative stress and thereby enhance immune activation, we introduced a FITC label onto the PC peptide to facilitate cellular‐level tracking of Ferritin‐PC@PBNZs. Fluorescence imaging experiments using the BMDCs confirmed the efficient internalization and lysosomal localization of the nanozyme vaccine. Colocalization analysis revealed that Ferritin‐PC@PBNZs exhibited a markedly higher degree of lysosomal internalization compared with the PC peptide and Ferritin‐PC groups (Figure 2d). Line‐scan analysis of LysoTracker Red and FITC signals revealed strong intracellular overlap, indicating that Ferritin‐PC@PBNZs predominantly localize to lysosome‐associated compartments in DCs (Figure S9). Notably, no detectable FITC fluorescence signal was observed in the PC peptide and Ferritin‐PC group, which may be attributable to rapid lysosomal escape of the free peptide following internalization or to the negatively charged ferritin surface that disfavors active DCs uptake. Ferritin‐PC@PBNZs exhibits a near‐neutral surface charge, which is more permissive for cellular uptake. Subsequently, FITC‐labeled nanomaterials, including PC peptide, Ferritin‐PC, and Ferritin‐PC@PBNZs, were intraperitoneally injected into mice to evaluate in vivo cellular uptake. After 48 h, intestinal lymph node cells were isolated and analyzed by FCM to assess the internalization of DCs and macrophages. Quantitative FCM results shows that, compared with the PC peptide or Ferritin‐PC groups, DCs exhibited a much higher internalization of Ferritin‐PC@PBNZs, with the proportion of CD11c^+^FITC^+^ double‐positive cells reaching approximately 30%, whereas the two groups showed only about 10% or less (Figure 2e). This indicates a stronger uptake capacity of DCs for Ferritin‐PC@PBNZs. Notably, macrophage uptake showed no significant difference among the formulations (Figure 2f), likely reflecting the intrinsically high phagocytic ability of macrophages. Collectively, these findings indicate a preferential uptake of Ferritin‐PC@PBNZs by DCs, underscoring the critical role of the ferritin–PBNZs assembly in facilitating efficient antigen delivery.

To corroborate internalization and subcellular localization, TEM was conducted on dendritic cells following treatment with Ferritin‐PC@PBNZs and PBNZs of defined sizes (3, 60, and 170 nm). TEM revealed size‐dependent cellular uptake of PBNZs (Figure 2g). Specifically, 3 nm PBNZs were taken up to a greater extent and were broadly distributed throughout the cytoplasm, with a fraction localized within lysosomal compartments (Figure S10). The 60 nm PBNZs were efficiently internalized by DCs and predominantly accumulated in lysosomes. By contrast, DCs exhibited substantially lower uptake of 170 nm PBNZs, with only sparse particles observed in the cytoplasm. Notably, the nano‐vaccine complex Ferritin‐PC@PBNZs, constructed from 60 nm PBNZs, was also efficiently taken up by DCs and localized mainly to lysosomes, as clearly visualized in the TEM images. These results are consistent with the previous lysosomal localization findings and further support the ROS‐mediated activation mechanism of DCs induced by PBNZs.

B‐cell and T‐cell activation is a prerequisite for the generation of high‐affinity, antigen‐specific antibodies. We next assessed whether this enhanced antigen delivery and DCs activation translated into stronger adaptive immune responses. Flow cytometric analysis of intestinal lymph node cells revealed that Ferritin‐PC@PBNZs treatment significantly increased the population of activated B cells (CD19^+^CD40^+^) compared with PBNZs, PC peptide, Ferritin‐PC, or PBS controls (Figure 2h,i). The upregulation of CD40, a key co‐stimulatory molecule required for B‐cell proliferation and class‐switch recombination, indicates efficient B‐cell activation and potential enhancement of antigen‐specific antibody responses. Similarly, Ferritin‐PC@PBNZs significantly elevated the proportion of activated T cells (CD3^+^CD69^+^) relative to control groups and PBNZs‐only (Figure 2j,k). CD69 reflects the rapid engagement and priming of T cells in response to antigen presentation by mature DCs. The concurrent activation of both B and T lymphocytes suggests that Ferritin‐PC@PBNZs effectively bridge innate and adaptive immunity, enabling robust humoral immune responses. In contrast, co‐injection of PC peptide and PBNZs without ferritin conjugation resulted in no significant enhancement in B‐cell activation. This phenomenon can be explained by the fact that, without the ferritin carrier, free peptides are more susceptible to rapid degradation and clearance by the mononuclear phagocyte system. Collectively, following internalization of PBNZs, the acidic lysosomal milieu and locally available H_2_O_2_ can promote POD‐like catalysis, generating short‐lived ROS within these compartments. This environment‐dependent redox signalling can drive DCs maturation by activating redox‐sensitive pathways, thereby promoting antigen‐presentation programmes. Specifically, it increases the expression of MHC II and the co‐stimulatory molecule CD86, and enhances the production of immunostimulatory cytokines, ultimately supporting efficient T‐cell priming. These results demonstrate that the Ferritin‐PC@PBNZs not only enhance DCs maturation and antigen uptake but also promote downstream activation of adaptive immune effectors in intestinal lymphoid tissues.

### 2.4 Ferritin‐PC@PBNZs Vaccination Elicits Strong Antigen‐Specific Humoral Immune Responses

To evaluate the immunogenicity of the Ferritin‐PC@PBNZs vaccine, mice were immunized with different formulations, including the PC peptide alone, Ferritin‐PC, PBNZs, and the Ferritin‐PC@PBNZs complex. All formulations were administered via intraperitoneal injection, with the primary immunization conducted on day 1 followed by a booster dose on day 10. Serum samples were collected at weeks 1, 2, and 4 after the initial immunization (Figure 3a). The levels of anti‐PC antibodies were subsequently quantified using an enzyme‐linked immunosorbent assay (ELISA) to monitor the kinetics of antibody production over time. As shown in Figure 3b, a significant IgM response was observed early after vaccination, indicating that the antigens exposed in the presence of Ferritin‐PC can activate the immune system, regardless of whether the PBNZs adjuvant was included. Notably, Ferritin‐PC@PBNZs elicited a stronger anti‐PC IgM response compared to the PC peptide alone, Ferritin‐PC, or PBNZs groups. The week‐specific data for total anti‐PC IgM concentrations are provided in Figure S11. Quantitative analysis of antibody titers provided key evidence for the strength of the humoral immune response induced by the Ferritin‐PC@PBNZs vaccine. Endpoint dilution assays revealed that the anti‐PC IgM antibody titers in the serum of mice vaccinated with Ferritin‐PC@PBNZs reached 1:10^5^, and even higher in some samples (Figure 3c).

Moreover, the inclusion of the nanozyme adjuvant PBNZs significantly enhanced the magnitude and persistence of the IgG response (Figure 3d; Figure S12). Compared to the adjuvant‐free Ferritin‐PC control group, the presence of PBNZs evidently boosted DCs activation and antigen presentation efficiency, thereby stimulating downstream B cells and T cells to initiate immune activation and produce higher levels of specific antibodies. In stark contrast, the IgG antibody titers in the control groups, including free PC peptide, Ferritin‐PC alone, and PBNZs adjuvant groups, plateaued at dilution concentrations of approximately 1:10^4^ (Figure 3e). These results demonstrate that the synergistic effect of the PBNZs nanozyme adjuvant and Ferritin‐PC antigen generates an antibody response that substantially surpasses conventional levels.

To further characterize the quality of the antibody response induced by the Ferritin‐PC@PBNZs vaccine, we performed a detailed analysis of the anti‐PC IgG subclasses in serum. We observed that vaccination with Ferritin‐PC@PBNZs induced a broad array of anti‐PC IgG subclass responses, including IgG1, IgG2b/c, and IgG3. Notably, levels of IgG2b (Figure 3f) and IgG2c (Figure 3g) were significantly elevated, suggesting that Ferritin‐PC@PBNZs preferentially induces a Th1‐type immune response. Among all IgG subclasses, IgG3 exhibits the strongest complement activation capacity and possesses the highest affinity for Fc receptors. As shown in Figure 3h, the strong IgG3 response elicited by the Ferritin‐PC@PBNZs vaccine indicates that the vaccine‐induced antibodies possess potent effector functions, capable of efficiently eliminating PC antigen–expressing damaged cells or pathogens through complement‐dependent cytotoxicity and antibody‐dependent cellular phagocytosis. This effector activity may contribute directly to therapeutic efficacy at inflammatory sites. In parallel, the detectable IgG1 response suggests that the vaccine also retains the ability to activate Th2‐associated pathways (Figure 3i), thereby ensuring sufficient B‐cell help and sustained antibody production. Such a balanced Th1/Th2 immune profile is likely to provide more comprehensive and durable immune protection than a single‐polarized response. Notably, the IgG2 predominance induced by the Ferritin‐PC@PBNZs vaccine parallels the natural protective immune pathways in humans, thereby reinforcing intrinsic protective mechanisms.

### 2.5 Ferritin‐PC@PBNZs Immunizations are Protective in Chronic Colitis

Building on the above findings that the Ferritin‐PC@PBNZs vaccine elicits a potent systemic immune response, we further evaluated its therapeutic potential in a dextran sulfate sodium (DSS)‐induced acute colitis model. This model was established by allowing mice to freely consume drinking water containing 3% DSS for five consecutive days, during which DSS directly damages colonic epithelial cells and disrupts mucosal barrier integrity, thereby triggering severe intestinal inflammation. Subsequently, the mice were provided with sterile drinking water for recovery, and therapeutic outcomes were assessed on day 10. In the DSS‐induced acute colitis model, the Ferritin‐PC@PBNZs vaccine exhibited a pronounced protective effect. Compared with the disease control groups treated with free PC peptides, Ferritin‐PC alone, or the PBNZs adjuvant, mice immunized with the Ferritin‐PC@PBNZs vaccine showed markedly attenuated body weight loss, with only a 3.8% reduction. The trend of weight change in this group closely resembled that of the positive control group treated with the anti‐inflammatory drug 5‐aminosalicylic acid (5‐ASA), characterized by a gradual weight decrease beginning on day 6, reaching a minimum on day 8, and followed by a slight recovery thereafter (Figure 4a). These findings suggest that vaccination with Ferritin‐PC@PBNZs confers a protective effect comparable to 5‐ASA in maintaining body weight during colitis progression. The disease activity index (DAI), which comprehensively evaluates changes in body weight, stool consistency, and the presence of fecal blood, serves as a direct indicator of disease severity. Throughout the experimental period, mice immunized with the Ferritin‐PC@PBNZs vaccine exhibited significantly lower DAI scores than those in the disease control group (Figure 4b). Specifically, vaccinated mice showed more formed stools without the profuse watery diarrhea commonly observed in control animals, and the incidence of fecal bleeding was markedly reduced. Although all DSS‐treated groups displayed characteristic body weight loss and cyclical fluctuations in DAI scores, the magnitude of these fluctuations was substantially attenuated in the vaccine‐immunized group. At the end of the 10‐day evaluation period, mice were euthanized, and the colon length was measured. Colon shortening serves as an intuitive morphological indicator of the severity of intestinal inflammation and fibrosis, with shorter colons reflecting more pronounced inflammation and tissue damage–induced fibrotic contraction. In the healthy control group, the average colon length was maintained at approximately 7–8 cm. Among the DSS‐treated groups, only the Ferritin‐PC@PBNZs and 5‐ASA–treated mice exhibited no apparent colon shortening, showing significantly longer colons compared with control groups and nearly comparable lengths to those of the healthy controls (Figure 4c,d). These findings clearly indicate that the Ferritin‐PC@PBNZs vaccine markedly alleviated the severity of colonic inflammation.

Histopathological analysis of colon tissues by hematoxylin and eosin (H&E) staining revealed pronounced morphological differences among the treatment groups (Figure 4e). In the PBS‐treated disease control mice, the colon exhibited typical features of DSS‐induced injury, including severe crypt distortion, detachment, and even complete loss of glandular architecture. The epithelial integrity was profoundly disrupted, characterized by extensive epithelial cell shedding, ulcer formation, and a marked reduction in goblet cell numbers. Moreover, dense inflammatory cell infiltration was observed in both the mucosal and submucosal layers, accompanied by evident tissue edema. Collectively, these pathological changes confirmed that the DSS model successfully induced severe disruption of colonic barrier function. In contrast, colonic tissues from mice immunized with the Ferritin‐PC@PBNZs vaccine retained a relatively intact intestinal barrier architecture. The crypt structures remained largely preserved, and the epithelial layer exhibited continuous integrity, closely resembling that of the healthy control group. These histological findings indicate that the Ferritin‐PC@PBNZs vaccine effectively protected the structural integrity of the intestinal barrier and mitigated inflammation‐mediated tissue damage in the DSS‐induced colitis model, demonstrating pronounced intestinal protective and reparative effects.

**FIGURE 5:**
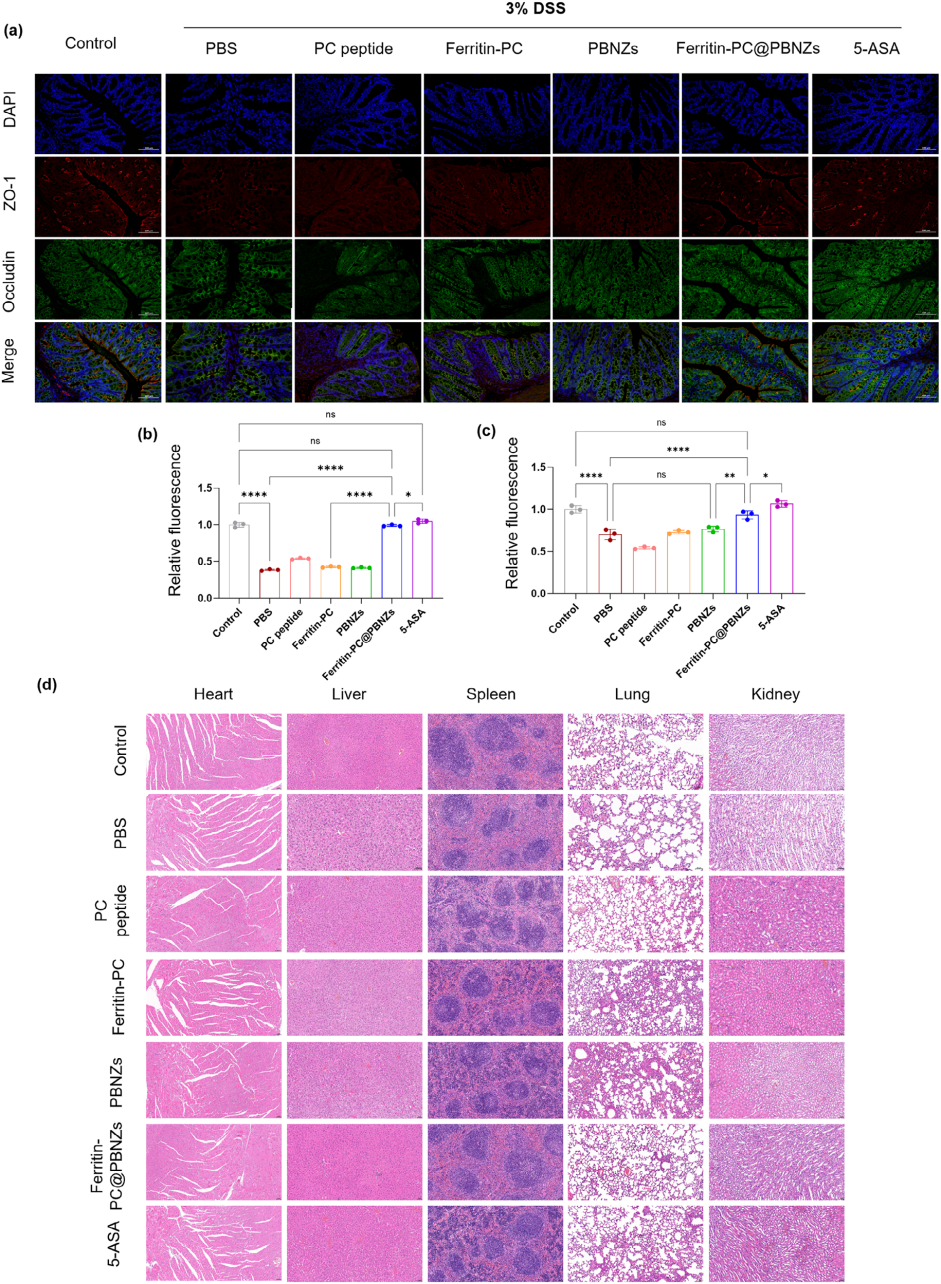
Protective effects of Ferritin‐PC@PBNZs immunization on the intestinal barrier and its biosafety evaluation in the acute colitis model. (a) Levels of the tight‐junction proteins ZO‐1 and occludin in colon sections after immunization. DAPI (blue), ZO‐1 (red), and occludin (green). Scale bar: 100 µm. (b) Quantitative analysis of ZO‐1 fluorescence intensity. The fluorescence intensity was normalized to the mean value of the control group. (c) Quantitative analysis of occludin fluorescence intensity. Data were normalized to the mean fluorescence intensity of the control group. One‐way ANOVA with Tukey's multiple comparison test for (b) and (d). *n* = 3, *p* < 0.05 (^*^), *p* < 0.01 (^**^), *p* < 0.001 (^***^), *p*<0.0001 (^****^), ns, not significant. (d) H&E analysis of the heart, liver, spleen, lung, and kidney.

Furthermore, immunofluorescence staining was performed to evaluate the expression and localization of the tight junction proteins ZO‐1 and occludin in colonic tissues, aiming to determine whether the alleviation of disease severity by Ferritin‐PC@PBNZs immunization in DSS‐induced colitis was associated with modulation of these junctional proteins. The results showed that DSS treatment markedly disrupted the continuity of tight junctions and reduced their expression levels, as evidenced by weakened and discontinuous fluorescence signals. In contrast, mice vaccinated with the Ferritin‐PC@PBNZs vaccine maintained a continuous, linear distribution of ZO‐1 and occludin along the intestinal epithelium, with significantly stronger fluorescence intensity than that observed in the disease control group (Figure 5a). The significant differences in the quantitative immunofluorescence data of the Ferritin‐PC@PBNZs‐treated group compared with the experimental groups support its role in promoting intestinal barrier restoration (Figure 5b,c). These findings collectively demonstrate, at both the morphological and molecular levels, that the nano‐vaccine promotes intestinal barrier repair in DSS‐induced colitis by specifically preserving and upregulating the structure and function of tight junction proteins.

Finally, H&E staining was performed on major organs, including the heart, liver, spleen, lungs, and kidneys, from all experimental groups to assess systemic safety. Histological examination revealed that the tissue architecture of these organs remained intact in vaccinated mice, with no observable signs of edema, inflammatory cell infiltration, degeneration, or necrosis (Figure 5d). These findings indicate that immunization with the Ferritin‐PC@PBNZs vaccine did not induce detectable systemic toxicity at the experimental dosage, demonstrating favorable biocompatibility and biosafety.

### 2.6 Ferritin‐PC@PBNZs Immunizations Alter Gut Microbiome Diversity

**FIGURE 6:**
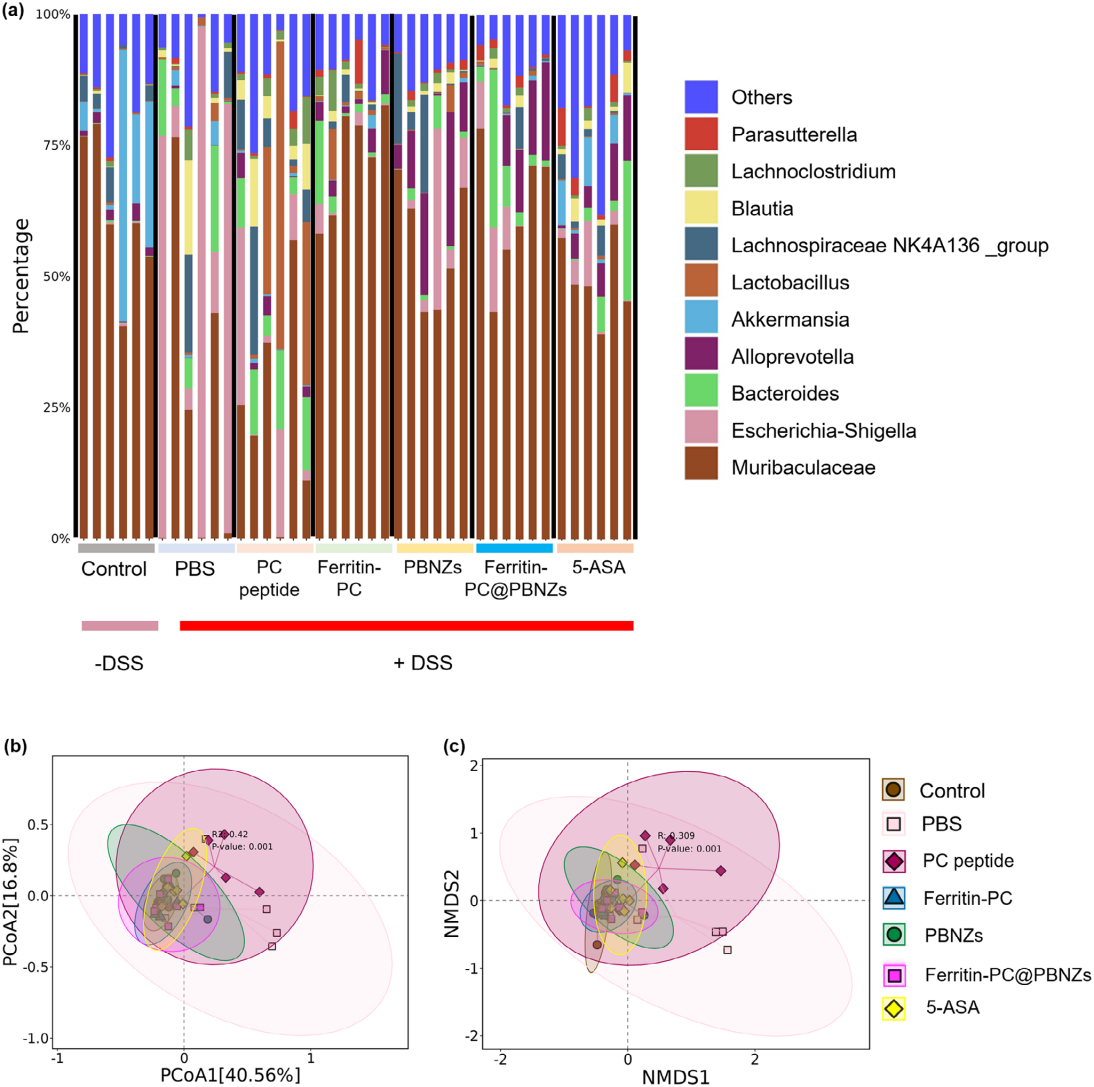
Ferritin‐PC@PBNZs reshapes the gut microbiome in the DSS‐induced colitis model. (a) Family‐level taxonomic histograms illustrating relative abundance of the gut microbiome (b) Distance matrix derived from the beta‐diversity analysis. (c) Non‐metric multidimensional scaling (NMDS) ordination analysis based on the distance matrix. Fecal samples were collected from mice during the final week before the end of the experiment and analyzed by sequencing the variable regions of the 16S rRNA gene.

Alterations in the gut microbiome have been linked to IBD and are also observed in DSS‐induced colitis. Therefore, we investigated whether immunization with Ferritin‐PC@PBNZs reshapes the gut microbiome in the DSS‐induced colitis model and further assessed the potential contribution of such changes to disease progression following colitis induction. Sequencing of the variable regions of the bacterial 16S rRNA gene from faecal samples revealed that DSS administration altered the relative abundance of multiple microbial families compared with healthy mice (Figure 6a), with a marked reduction in Akkermansia. In the acute colitis model, the PBS‐treated group, which exhibited the most severe clinical symptoms, showed a high abundance of Escherichia–Shigella, suggesting that this taxon may be associated with the extent of intestinal injury. Notably, compared with the PBS group, both the PBNZs‐treated group and the Ferritin‐PC@PBNZs immunized group exhibited an increased abundance of Alloprevotella. However, this increase did not translate into an attenuation of colitis severity in the PBNZs‐treated group. We therefore infer that the enrichment of Alloprevotella is attributable to PBNZs rather than to Ferritin‐PC. We further characterized DSS‐associated alterations in the gut microbiota using beta‐diversity analysis. Both the distance matrix (Figure 6b) and non‐metric multidimensional scaling (NMDS) ordination based on this matrix (Figure 6c) showed that the microbiota composition of DSS‐challenged mice in the PBS‐treated and PC peptide–treated groups was clearly distinct from that of the treatment groups. Although the Ferritin‐PC@PBNZs immunized group also differed from the DSS‐induced colitis model, it exhibited partial similarity to the healthy control and 5‐ASA–treated groups. Together, these results indicate that Ferritin‐PC@PBNZs immunization partially counteracts DSS‐induced dysbiosis, shifting the microbial community towards a healthier‐like configuration. This microbiota remodelling may contribute to the improved intestinal inflammatory outcomes observed in the treated mice.

## 3 Conclusion

In summary, we developed an immunotolerance strategy based on PC‐vaccine adjuvanted with PBNZs for the treatment of IBD. This strategy leverages the ferritin carrier displaying PC epitopes that enhance antigen stability and presentation. Additionally, PBNZs further increase the density of antigen and lysosomal retention, where the POD‐like catalytic activity promotes the activation and maturation of DCs, driving a sustained and specific antibody response that alleviates inflammation and protects the intestinal barrier. We found that the synergistic interaction between the PBNZs adjuvant and the Ferritin‐PC antigen elicited a remarkably strong antibody response, inducing broad anti‐PC IgG subclass production, including IgG1, IgG2b/c, and IgG3. The Ferritin‐PC@PBNZs vaccine not only favored a Th1‐skewed immune response but also retained the capacity to activate Th2‐associated pathways, forming a balanced Th1/Th2 immune profile that may provide more comprehensive and durable protection than a single‐polarized response. In the DSS‐induced colitis model, vaccination with Ferritin‐PC@PBNZs markedly alleviated disease severity, accompanied by elevated anti‐PC antibody levels and enhanced expression of tight junction proteins that reinforced intestinal barrier integrity. Collectively, these findings demonstrate that this immunotolerance‐inducing strategy, by harnessing natural antibody responses, offers a promising approach to overcome current therapeutic limitations in IBD treatment.

## 4 Experimental Section

### 4.1 Ethics Statement

All the mouse experiments carried out in this study were approved by the Southeast University Animal Care and Use Committee (approval number: 20210526003) and followed the National Institutes of Health Guide for the Care and Use of Laboratory Animals.

### 4.2 Materials

All chemical reagents were purchased from Sigma‐Aldrich unless wise stated. Antibodies used for flow cytometry and immunofluorescence were listed in Tables S1 and S2, respectively.

### 4.3 Synthesis PBNZs and PBNZs‐PAH

According to previous reports, 60 nm PBNZs were synthesized using a conventional method [60]. Briefly, 8 g of polyvinylpyrrolidone (PVP, K30) and 0.696 g of potassium ferricyanide (K_3_[Fe(CN)_6_]) were dissolved in 40 mL of distilled water and magnetically stirred for 30 min in the presence of 1 M HCl. The mixture was then transferred to an oil bath and heated at 100°C for 24 h. After synthesis, the obtained PBNZs were purified using ultrafiltration tubes (MWCO: 100 kDa, Millipore, USA) with distilled water for four cycles, 30 min each. The purified PBNZs were then dispersed in distilled water and stored at 4°C for further use. For surface modification, PBNZs were mixed with poly(allylamine hydrochloride) (PAH) at a molar ratio of 1:10 and stirred overnight at room temperature. After the reaction, the resulting product was washed three times with distilled water using 100 kDa ultrafiltration tubes for 30 min per wash to obtain PBNZs–PAH.

### 4.4 Ferritin Expression and Purification

The recombinant plasmid encoding SnoopCatcher‐tagged engineered ferritin was constructed and synthesized by GENEWIZ (China). The recombinant plasmid carrying the target protein gene was transformed into E. coli BL21 (DE3) cells (Vazyme, China) for protein expression in LB medium (1% NaCl, 1% tryptone, 0.5% yeast extract, and 0.1 mg/mL kanamycin). The culture was incubated at 37°C and 200 rpm until the optical density at 600 nm (OD_600_) reached 0.6–0.8. Protein expression was then induced with 0.6 mM isopropyl β‐D‐1‐thiogalactopyranoside (IPTG) and maintained at 25°C for 20 h. Cells were harvested by centrifugation at 5000 × g for 15 min at 4°C and resuspended in 50 mM pre‐cooled phosphate‐buffered saline (PBS). The bacterial cells were collected by centrifugation and disrupted by ultrasonication on ice to obtain cell lysates. The lysate was centrifuged again to collect the supernatant, which was subsequently incubated in a 70°C water bath for 15 min to denature most non–heat‐stable contaminant proteins. After high‐speed centrifugation for 10 min to remove the precipitate, the supernatant was loaded onto a HisTALON gravity column (Takara Bio, USA) for purification of the target protein. The protein concentration was determined using a BCA protein assay kit, and the size and purity of ferritin were analyzed by sodium dodecyl sulfate–polyacrylamide gel electrophoresis (SDS–PAGE) on 12% gels.

### 4.5 Conjugation of Phosphorylcholine with Peptides

Peptide sequences are listed in Figure S1. All peptides were synthesized by GENEWIZ Corporation (Suzhou, China). The lyophilized peptides were dissolved in sterile water at a concentration of 1 mg/mL and mixed with 40 equivalents of 2‐(methacryloyloxy)ethyl‐2‐(trimethylammonio)ethyl phosphate in aqueous solution. The reaction mixture was stirred overnight at room temperature. Unreacted PC molecules were removed by ultrafiltration using 3 kDa centrifugal filters with sterile 1× PBS as the washing buffer (30 min per cycle, repeated five times). The resulting PC–peptide conjugates were confirmed by MALDI‐TOF mass spectrometry. The purified conjugates were collected and stored at −20°C until use. Peptides containing fluorescent labels were prepared following the same procedure.

### 4.6 Characterization of Ferritin‐PC@PBNZs

Morphology, particle size, and zeta potential. The particle size and morphology of the Ferritin‐PC@PBNZs were analyzed using transmission electron microscopy (TEM) (Tecnai G2, FEI, USA). Dynamic Light Scattering (DLS) (ZEN 3600, Malvern, UK) was employed to measure the hydrodynamic size and zeta potential.

### 4.7 POD‐Like Activity Assays

Different sizes of PBNZs or Ferritin‐PC@PBNZs were tested at room temperature using TMB as substrates in the presence of H_2_O_2_ in a 0.2 M HAc‐NaAc buffer solution at pH 4.6. The absorbance of the color reaction at 650 nm was measured at specific time points using a SpectraMax Absorbance Reader (CMAx Plus, Molecular devices, USA) to quantify the POD‐like activity. Reaction systems comprised 2 µL of 10 µg/mL PBNZs, 20 µL of 30% H_2_O_2_, 10 µL of 10 mg/mL TMB, and pH 3.6 buffer solutions, totaling 200 µL.

### 4.8 Evaluation of Intracellular GSSG/GSH Ratio

Approximate 5 ×10ˆ^5^ BMDCs were seeded in a 6‐well plate and cultured overnight. Subsequently, the cells were treated with different sizes of PBNZs or Ferritin‐PC@PBNZs for 24 h, after which they were harvested and lysed. The levels of GSSG and GSH were assessed using a fluorimetric glutathione GSH/GSSG assay kit (AAT Bioquest, 10056) following the manufacturer's protocols.

### 4.9 Subcellular localization of Ferritin‐PC@PBNZs

For subcellular distribution analysis, primary DCs were isolated from mouse bone marrow and cultured with GM‐CSF (20 ng/mL) for 4 days to induce differentiation. The cells were then incubated with FITC‐labeled Ferritin‐PC@PBNZs, Ferritin‐PC, or PC peptide for 24 h. After incubation, the cells were washed with PBS and stained with LysoTracker (Thermo Fisher Scientific) at 37°C for 40 min to visualize lysosomes, followed by nuclear staining with Hoechst 33342 at room temperature for 10 min. After final washing with PBS, fluorescence images were acquired using a laser scanning confocal microscope (FV3000, Olympus, Japan). Colocalization between Ferritin‐PC@PBNZs and lysosomes was analyzed based on the merged fluorescence signals.

In addition, BMDCs were incubated with PBNZs of different sizes or Ferritin‐PC@PBNZs for 24 h, then collected and fixed in 2.5% glutaraldehyde for TEM imaging. The fixed cells were subsequently dehydrated, embedded, polymerized, and sectioned into ultrathin slices to examine material internalization.

### 4.10 Flow Cytometric Analysis of Immune Cell Activation

Mice were intraperitoneally injected with Ferritin‐PC@PBNZs, Ferritin‐PC, PC peptide, or PBNZs at a dose of 10 mg/kg. After 48 h, intestinal lymph node cells were isolated and collected using cell staining buffer (BioLegend). According to the manufacturer's instructions, the cells were stained for 30 min with the following antibodies: CD11c‐PE, F4/80‐APC, CD19‐PE, CD40‐APC, CD3‐PE, and CD69‐Cy5.5. Flow cytometric data were acquired on a Beckman Coulter Cytoflex LX (Beckman Coulter, USA) and analyzed using FlowJo software.

### 4.11 Detection of Anti‐PC Antibody Responses via ELISA

Anti‐PC antibody responses in serum collected from the mouse tail vein blood were measured by ELISA. Briefly, plates were coated with 200 µg ml^−1^ PC with 1% BSA in 1X PBS at 4°C overnight. Before adding serially diluted mouse serum samples, the ELISA plates were blocked with SuperBlock blocking buffer solution (Thermo Fisher, 37515) for 1 h. After serum incubation at 37°C for 5 h, the plates were washed with PBST (0.05% Tween‐20 in PBS). HRP‐conjugated anti‐mouse IgM, IgG, IgG1, IgG2b, IgG2c or IgG3 antibodies (Abcam), diluted according to the manufacturer's instructions, were then added and incubated for 1 h. After additional washes with PBST, TMB substrate solution (Millipore) was added, followed by stop solution. Absorbance was measured at 450 nm. Antibody titres were defined using an absorbance cut‐off above background of 0.2 optical density (OD), where titre 3 represents an OD value greater than 0.2 at 1:10^3^ dilution and so on up to titre 6 at 1:10^6^ dilution.

### 4.12 DSS‐Induced Colitis and Assessment

All experiments were conducted using 6–10‐week‐old male C57BL/6 mice. The mice were housed under a standard 12‐h light/12‐h dark cycle at an average ambient temperature of 25°C. All animals were purchased from GemPharmatech Co., Ltd. (Jiangsu, China). After one week of acclimation, mice were randomly divided into seven groups (*n* = 6 per group). A PBS without DSS group served as the healthy control, and these mice were housed separately and supplied with normal drinking water throughout the experiment. The immunization and booster schedule is shown in Figure 3a. Twenty days after the initial vaccination, an acute colitis model was induced by administering 3% (w/v) DSS (molecular grade, MP Biomedicals, 160110) in drinking water for 5 days, followed by 5 days of normal water. Disease activity index (DAI) scores were determined daily and were based on body weight loss (as a percentage of initial weight; ≥100% = 0, 90–99% = 1, 80–89% = 2, <80% = 3), fecal occult blood (0 = none, 3 = present), and stool consistency (0 = normal, 1 = soft, 2 = very soft, 3 = diarrhea), with a maximum daily score of 9. If no fecal sample was available for a given mouse on a given day, the DAI score for that day was excluded. At the study endpoint, mice were euthanized, and the colon length was measured from the cecum base to the rectum.

### 4.13 Histopathology and Immunofluorescence

At the end of the DSS‐induced colitis experiment, mice were euthanized, and major organs including the heart, liver, spleen, lungs, kidneys, and mid‐colon, were collected. Tissues were fixed in 4% paraformaldehyde solution for 24 h, embedded in paraffin, sectioned, and stained with hematoxylin and eosin (H&E) for histopathological examination. For immunofluorescence analysis, colon tissues from each mouse were stained for the tight junction proteins ZO‐1 and occludin. Immunofluorescence experiments were performed by Servicebio Biotechnology Co., Ltd. (Wuhan, China) following standard protocols.

### 4.14 Microbiome Analysis

Fecal samples were collected from mice during the final week before the end of the experiment and analyzed by sequencing the variable regions of the 16S rRNA gene. Microbial diversity was profiled on the DNBSEQ‐G99 sequencing platform using paired‐end sequencing of short‐insert libraries. Raw reads were merged and quality‐filtered, followed by clustering or denoising. Taxonomic assignment and relative abundance analyses were then performed, together with β‐diversity analysis. The denoising of the quality‐controlled data was performed using the DADA2 method within QIIME2 (v2024.10.1). The default threshold for filtering ASVs was set to 0.005% of the total number of sequences. The species reference databases used were the latest versions of SILVA, Greengenes, and Unite. Taxonomic assignment was performed using the Naive Bayes classifier. Microbiome analysis was performed by Servicebio Biotechnology Co., Ltd. (Wuhan, China) following standard protocols.

### 4.15 Statistical Analysis

Statistical analyses were performed using GraphPad Prism software. Means +, −, or ± s.e.m. are shown as specified in figure captions along with sample sizes. For multiple groups, one‐way ANOVA, two‐way ANOVA, or two‐way repeated measures ANOVA with Tukey's multiple comparison test were used. *p* < 0.05 (^*^), *p* < 0.01 (^**^), *p* < 0.001 (^***^), *p*<0.0001 (^****^), ns, not significant. Further, a mixed‐effects analysis was used for mouse weights and disease activity index data. Mixed‐effects analysis was used for the longitudinal (repeated‐measures) body weight and DAI data, with treatment and time as fixed effects and individual mice as a random effect; Tukey's multiple‐comparison test was applied for post hoc group comparisons.

## Author Contributions

Jingyi Sheng and Yubo Huang contributed equally to this work. J.S. and Y.H. data acquisition, analysis, investigation, and writing – original draft. X.L. and Y.L., data acquisition, investigation, Y.W. and Y.Z. validation, software, methodology, N.G. writing – review and editing, validation, supervision, resources, project administration, funding acquisition, conceptualization.

## Conflicts of Interest

The authors declare no conflicts of interest.

## Supporting information




**Supporting File**: advs73760‐sup‐0001‐SuppMat.docx.

## Data Availability

The data that support the findings of this study are available from the corresponding author upon reasonable request.;
