# Supplementary material for: A Prussian Blue Nanozyme‐Adjuvanted Vaccine Presenting Phosphocholine Antigens for Induction of Immunotolerance in Inflammatory Bowel Disease
Source: Adv Sci (Weinh). 2026 Jan 22;13(16):e22027. doi: 10.1002/advs.202522027 (PMC13042549; doi:10.1002/advs.202522027)
Supplement: Supplementary file 1 — Supporting File: advs73760‐sup‐0001‐SuppMat.docx. [file ADVS-13-e22027-s001.docx]

A Prussian Blue Nanozyme-Adjuvanted Vaccine Presenting Phosphocholine Antigens for Induction of Immunotolerance in Inflammatory Bowel Disease

Jingyi Sheng^1#^*, Yubo Huang^1#^, Xinyue Li^1^, Yin Liu^2^, Yuehuang Wu^3^, Yuxin Zhang^1^, Ning Gu^1,4^*

^1^ Jiangsu Key Laboratory for Biomaterials and Devices, School of Biological Sciences & Medical Engineering, Southeast University, Nanjing, 210009, China

^2^ Key Laboratory for Bio-Electromagnetic Environment and Advanced Medical Theranostics, School of Biomedical Engineering and Informatics, Nanjing Medical University, Nanjing 211166, China

^3^ School of Chemistry and Chemical Engineering, Southeast University, Nanjing, Jiangsu 210009, China

^4^ Engineering Medicine Research Group, and Nanjing Research Center for Biomedical Electron Microscopy (NRC-BEM), Medical School, Nanjing University, Nanjing 210093, China

#These authors contributed equally to this work.

* Corresponding authors: shengjingyi@seu.edu.cn, guning@nju.edu.cn

Tables S1. Antibodies used list.

| Antibody | Supplier | Catalog number |
| --- | --- | --- |
| HRP-conjugated Fcγ fragment-specific goat anti-mouse IgG | Jackson Immuno Research | 15- 035-071 |
| Goat Anti-Mouse IgM-HRP | Southern Biotech | 1021-05 |
| HRP-conjugated goat anti-mouse IgM | Southern Biotech | 5300-05B |
| HRP-conjugated goat anti-mouse IgG1 | Southern Biotech | 5300-05B |
| HRP-conjugated goat anti-mouse IgG2b | Southern Biotech | 5300-05B |
| HRP-conjugated goat anti-mouse IgG2c | Southern Biotech | 5300-05B |
| HRP-conjugated goat anti-mouse IgG3 | Southern Biotech | 5300-05B |
| F4/80-APC (Clone BM8) | BioLegend | 123116 |
| CD11c-PE (Clone N418) | BioLegend | 117310 |
| CD86-APC (Clone PO3) | BioLegend | 105114 |
| CD40-APC (Clone 3/23) | BioLegend | 124612 |
| CD19-PE (Clone N418) | BioLegend | 117309 |
| CD3-PE (Clone 17A2) | BioLegend | 100205 |
| CD69- PerCP/Cyanine5.5 (Clone H1.2F3) | BioLegend | 104521 |


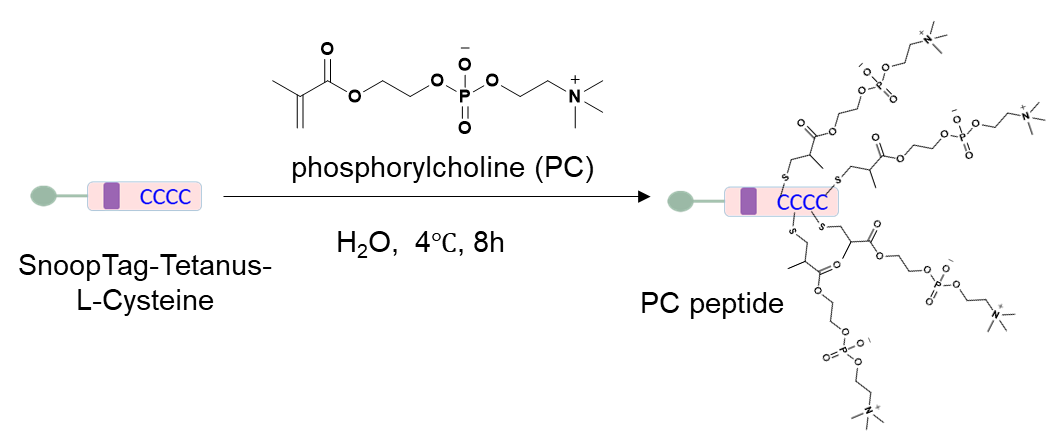


Supplementary Synthesis 1. Synthesis of PC peptide.


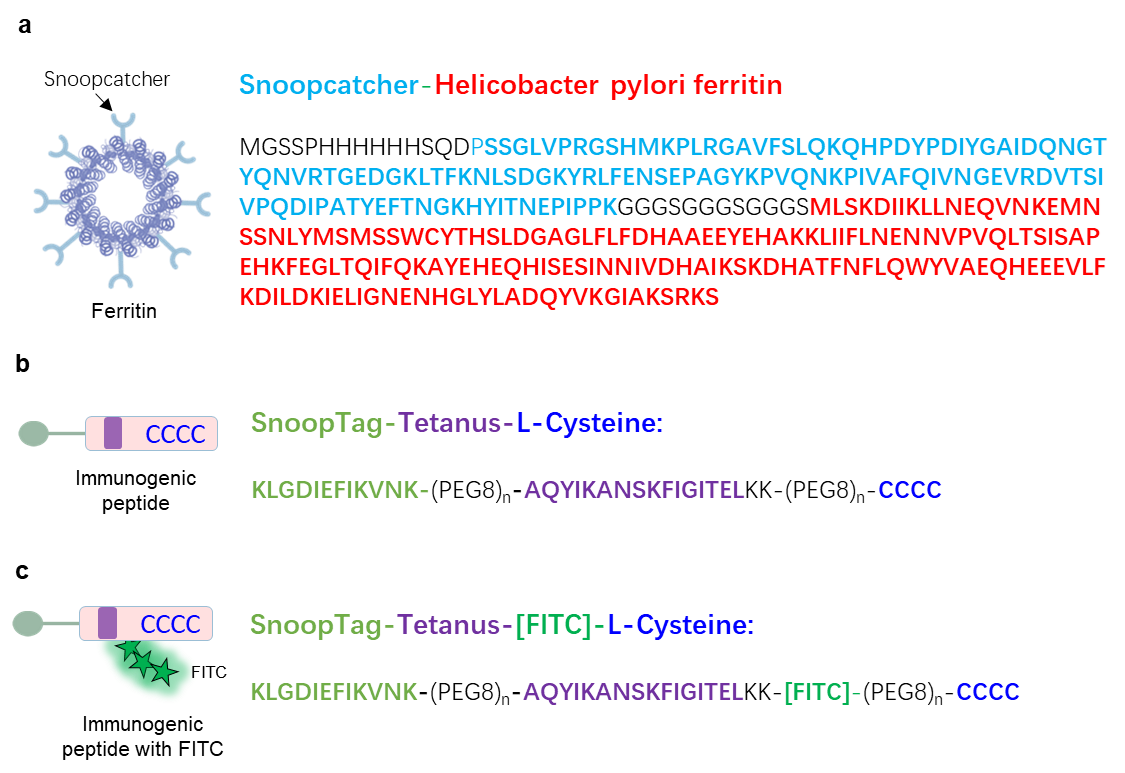


Figure S1. Ferritin and peptide investigated. (a) Sequence design of engineered ferritin carrying SnoopCatcher. (b) Design of the immunogenic peptide incorporating SnoopTag, a universal tetanus-toxoid T-cell epitope, and four C-terminal cysteine. (c) Immunogenic peptide with FITC label.


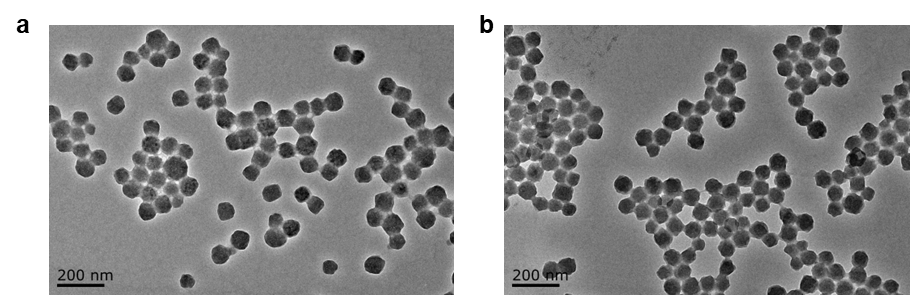


Figure S2. TEM images of PBNZs (a) and PB-PAH (b). Scale bar: 200 nm.


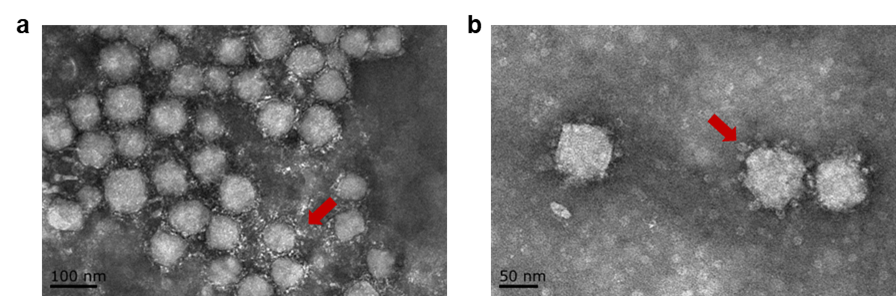


Figure S3. Negative-staining TEM images of Ferritin-PC@PBNZs at different magnifications: 100 nm (a) and 50 nm (b).


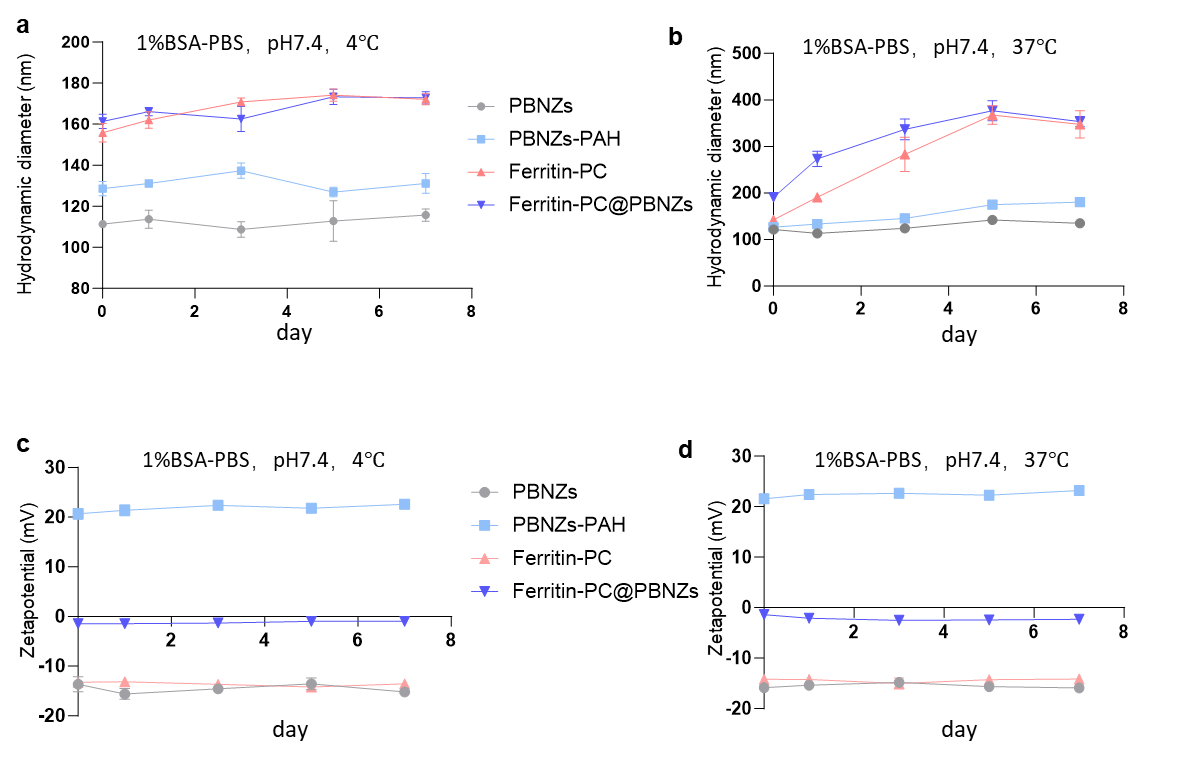


Figure S4. The stability of PBNZs, PBNZs-PAH, Ferritin-PC, and Ferritin-PC@PBNZs nanomaterials incubated in PBS (pH 7.4) containing 1% BSA at 4 °C and 37 °C. (a, b) Hydrodynamic diameter was monitored by dynamic light scattering (DLS) (a, 4 °C; b, 37 °C). (c, d) Zetapotential was determined by electrophoretic light scattering (c, 4 °C; d, 37 °C) to evaluate time-dependent size evolution and surface charge stability under protein-rich conditions.


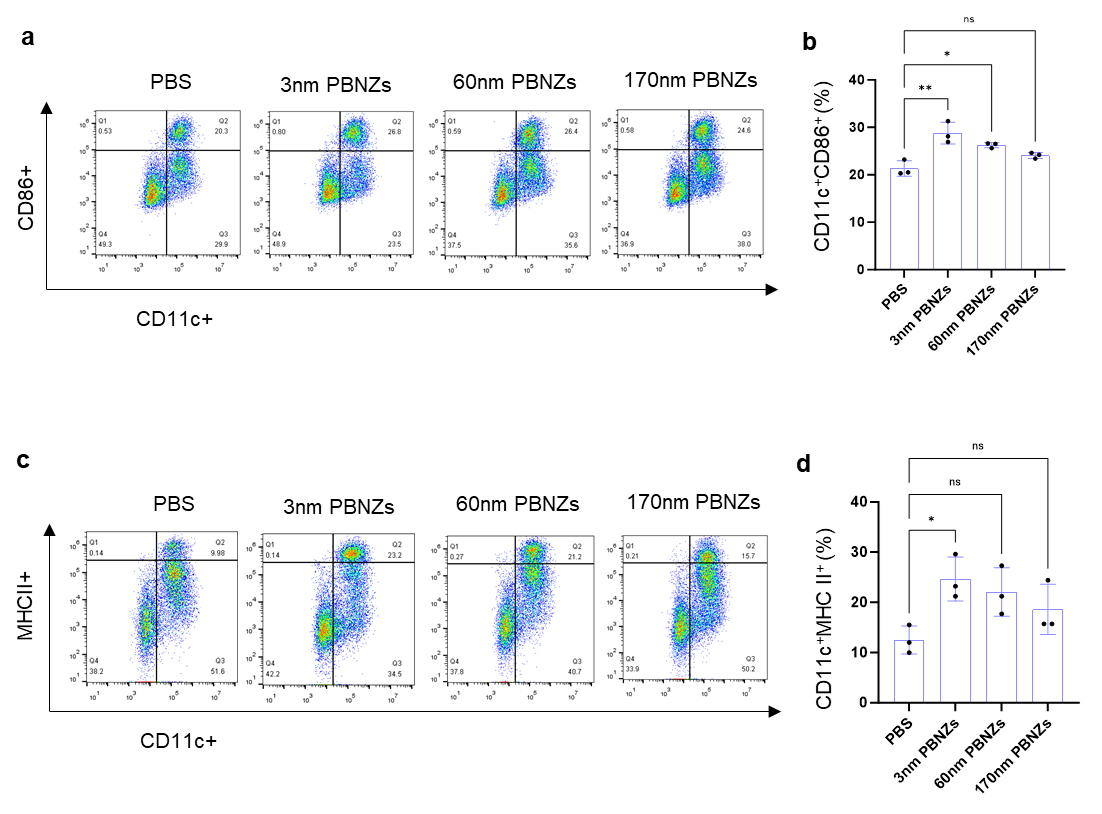


Figure S5. Flow cytometric (FCM) analysis of bone marrow–derived dendritic cells (BMDCs) activation. After treating with PBNZs of different sizes for 24 hours, staining with anti-CD11c and anti-CD86 or anti-MHCII antibodies was performed and analyzed by FCM. (a) FCM analysis of DCs with CD11c^+^CD86^+^. (b) FCM quantification of the percentage of CD11c^+^CD86^+^. (c) FCM analysis of DCs with CD11c^+^MHCII^+^. (b) FCM quantification of the percentage of CD11c^+^MHCII^+^. Differences were considered statistically significant when P < 0.05 (*), P < 0.01 (**), P < 0.001 (***), P<0.0001 (****). ns, not significant.


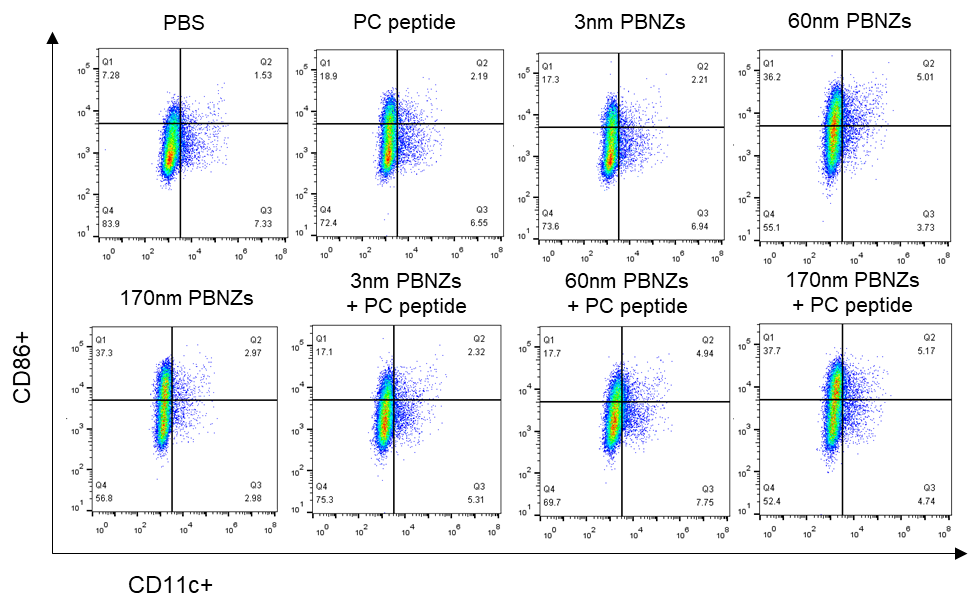


Figure S6. FCM analysis of DCs activation. Mice were intraperitoneally injected with various sizes of PBNZs alone or a physical mixture of PBNZs and PC peptide. After 48 hours, cells from intestinal lymph nodes were harvested and analyzed by flow cytometry following staining with anti-CD11c and anti-CD86 antibodies.


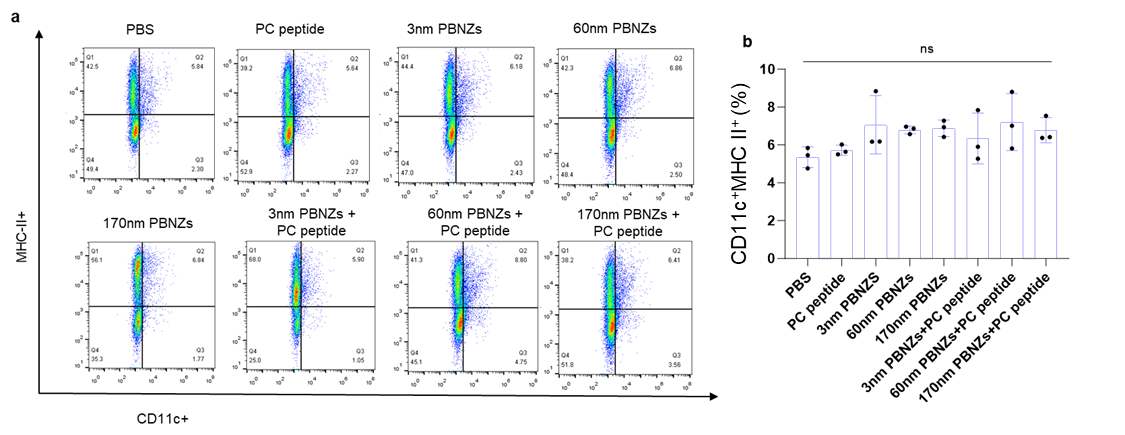


Figure S7. FCM analysis of dendritic cells DCs activation. Mice were intraperitoneally injected with various sizes of PBNZs alone or a physical mixture of PBNZs and PC peptide. After 48 hours, cells from intestinal lymph nodes were harvested and analyzed by flow cytometry following staining with anti-CD11c and anti-MHCII antibodies. (a) FCM analysis of DCs. (b) FCM quantification of the percentage of CD11c^+^MHCII^+^. ns, not significant.


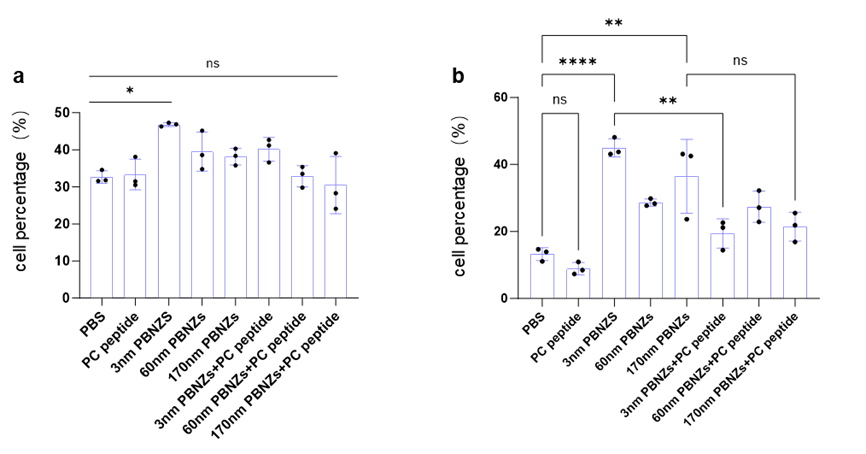


Figure S8. FCM analysis of B and T cell activation. Similar to the procedure described in Fig. S7, the collected intestinal lymph node cells were stained with antibodies. (a) FCM analysis quantification of the percentage of CD19^+^CD40^+^ of B cell. (b) FCM analysis quantification of the percentage of CD3^+^CD69^+^ of T cell. Differences were considered statistically significant when P < 0.05 (*), P < 0.01 (**), P < 0.001 (***), P<0.0001 (****). ns, not significant.

Figure S9. Line-scan fluorescence quantitative analysis of the colocalization between Ferritin-PC@PBNZs and lysosomes. DCs were stained with LysoTracker Red to label lysosomes (red channel) and incubated with FITC-labeled Ferritin-PC@PBNZ (green channel). Fluorescence intensities in both channels were extracted along a selected linear ROI within the cell using ImageJ, and intensity profiles versus distance (a.u.) were plotted. The red and green signals showed a synchronous increase and highly overlapping peak distributions within the cellular region, indicating that Ferritin-PC@PBNZs are mainly localized in lysosome-associated compartments.


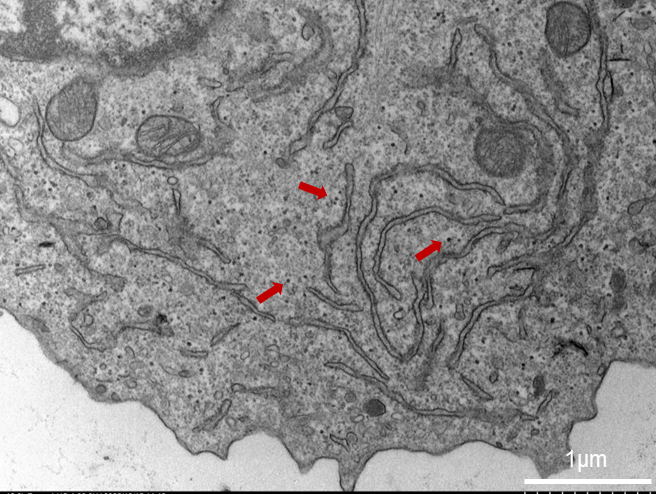


Figure S10. TEM images of BMDCs phagocytosing 3nm PBNZs. Scale bar: 1 μm.


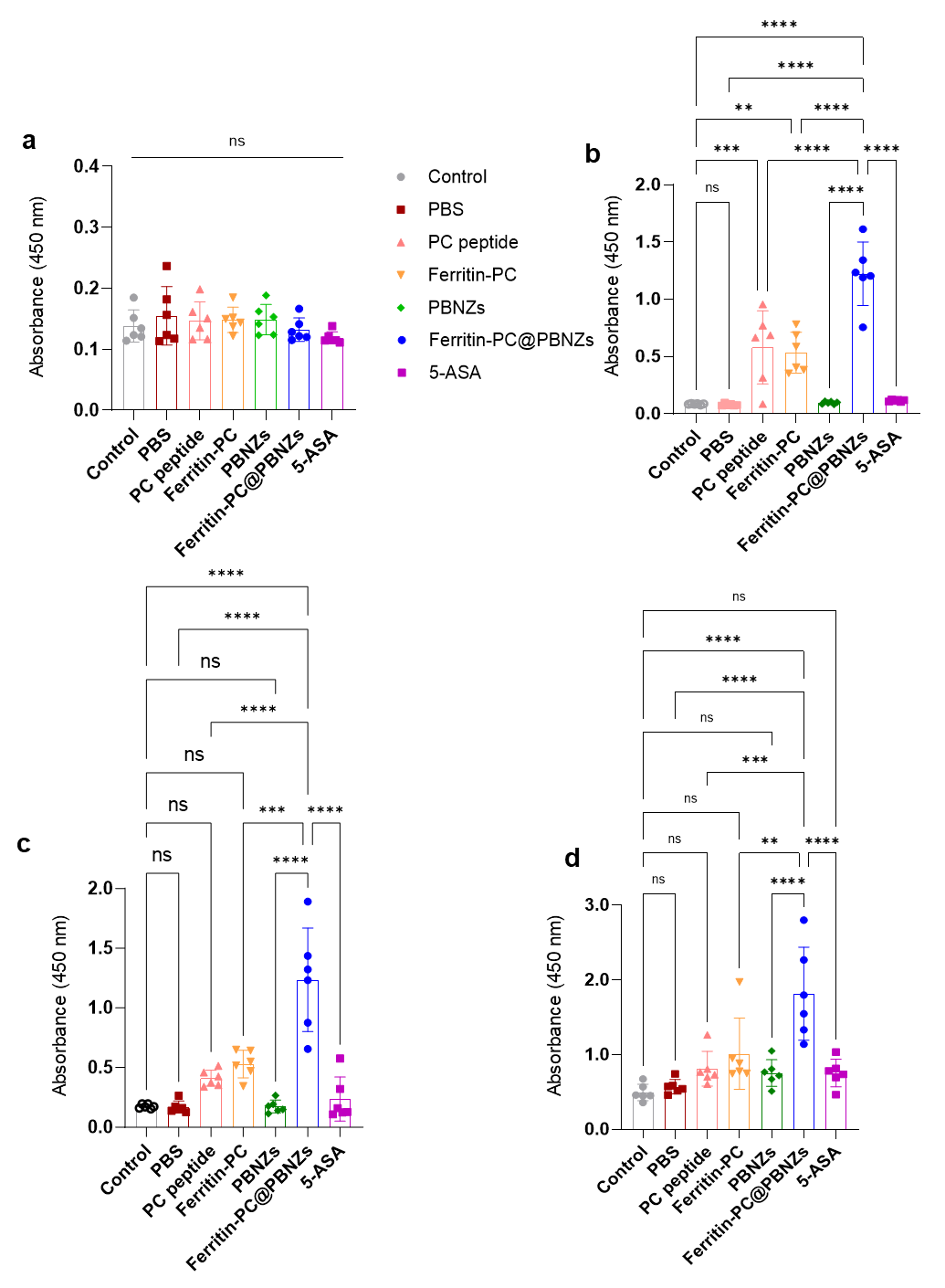


Figure S11. IgM antibody responses in serum samples collected at different time points: (a) week 0 (pre-immunization), (b) week 1, (c) week 2, and (d) week 4. All serum samples were diluted 1:1000 prior to testing. one-way ANOVA with Tukey’s multiple comparison test. P < 0.05 (*), P < 0.01 (**), P < 0.001 (***), P<0.0001 (****), ns, not significant.


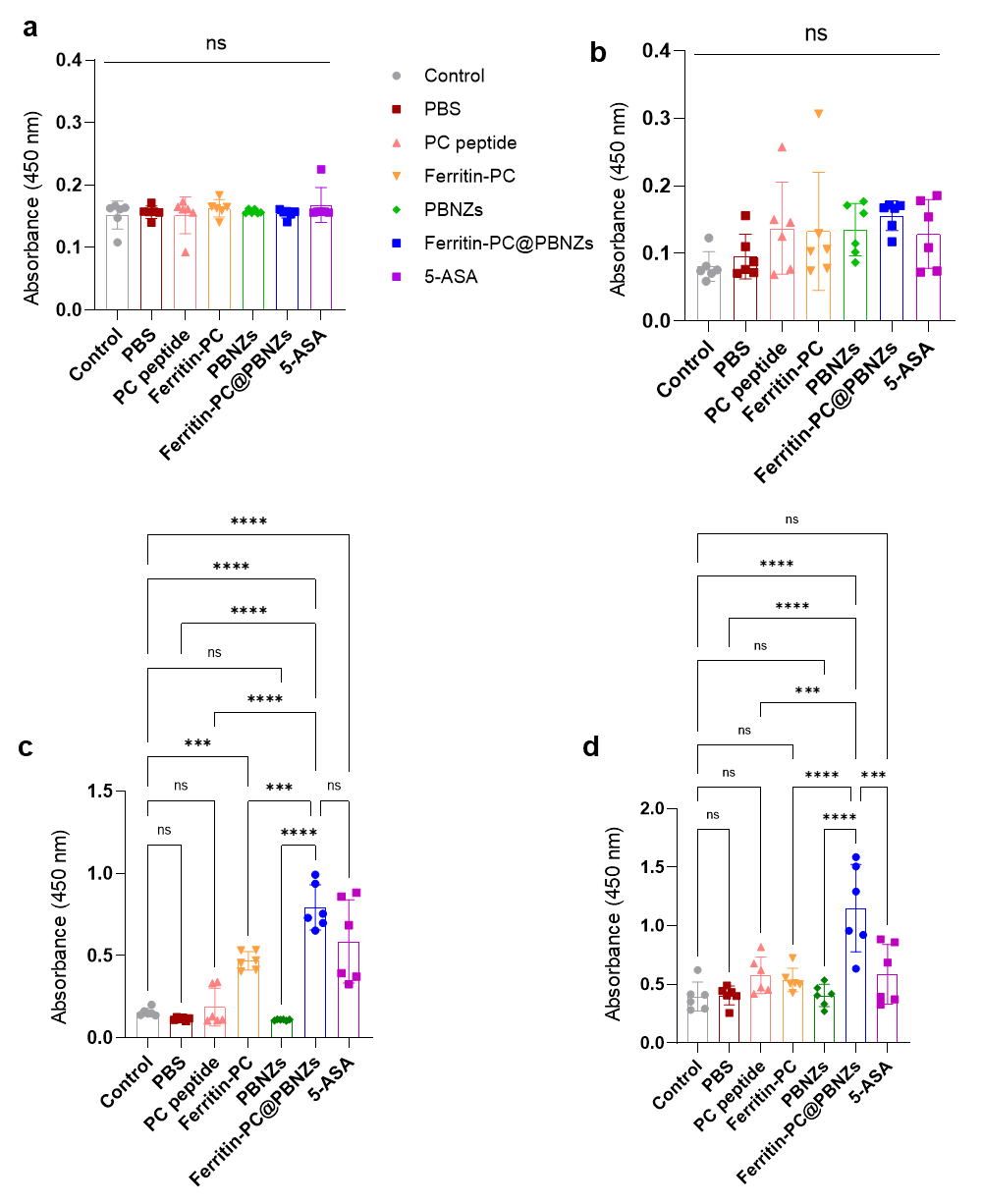


Figure S12. IgG antibody responses in serum samples collected at different time points: (a) week 0 (pre-immunization), (b) week 1, (c) week 2, and (d) week 4. All serum samples were diluted 1:1000 prior to testing. one-way ANOVA with Tukey’s multiple comparison test. P < 0.05 (*), P < 0.01 (**), P < 0.001 (***), P<0.0001 (****), ns, not significant.
